# Microbiome and Response to Therapy in Triple Negative Breast Cancer: A Systematic Review

**DOI:** 10.32604/or.2026.074215

**Published:** 2026-05-21

**Authors:** Mariana Lopes, Carlos Vila Nova, Rui Caetano Oliveira, Fernando Schmitt, Fernando Mendes, Diana Martins

**Affiliations:** 1 Escola Superior de Tecnologia da Saúde de Coimbra (ESTESC), Polytechnic University of Coimbra, Coimbra, Portugal; 2 Surgery Unit, Unidade Local de Saúde do Baixo Mondego, Figueira da Foz, Portugal; 3 Faculty of Medicine, University of Coimbra, Coimbra, Portugal; 4 Centro de Anatomia Patológica Germano de Sousa, Coimbra, Portugal; 5 RISE-Health, Department of Pathology, Medical Faculty of Porto University, Porto, Portugal; 6 H&TRC—Health & Technology Research Center, Coimbra Health School, Polytechnic University of Coimbra, Coimbra, Portugal; 7 Coimbra Institute for Clinical and Biomedical Research (iCBR) Area of Environment Genetics and Oncobiology (CIMAGO), Biophysics Institute of Faculty of Medicine, University of Coimbra, Coimbra, Portugal; 8 Center for Innovative Biomedicine and Biotechnology (CIBB), University of Coimbra, Coimbra, Portugal; 9 European Association of Biomedical Scientists, Brussels, Belgium

**Keywords:** Triple negative breast neoplasms, microbiota, chemotherapy, pathological complete response, immunotherapy

## Abstract

**Objectives:** Triple-negative breast cancer (TNBC) accounts for approximately 15% of all invasive breast cancers and is characterized by aggressive behavior, limited therapeutic options, and poor clinical outcomes. Due to the absence of hormone receptors and HER2 expression, systemic treatment relies predominantly on chemotherapy, which is associated with high rates of early recurrence and mortality. Emerging evidence suggests that alterations in the microbiome can contribute to TNBC progression and influence therapeutic response, particularly affecting the efficacy of chemotherapy and immunotherapy through immune-mediated mechanisms; however, its role in TNBC remains incompletely understood. This systematic review aims to explore the role of the microbiome in TNBC. It specifically aims to understand if the microbiome influences complete pathological response in TNBC. **Methods:** This systematic review was conducted in accordance with the Preferred Reporting Items for Systematic Reviews and Meta-Analyses (PRISMA) guidelines. A comprehensive literature search was performed in PubMed and Cochrane databases. Fourteen eligible studies were included, encompassing preclinical and clinical evidence. **Results:** The findings indicate that both gut and tumor-associated microbiota significantly influence therapeutic response in TNBC, especially in the context of neoadjuvant chemotherapy (NACT) and immune checkpoint blockade (ICB). Higher microbial diversity and the presence of specific commensal taxa were consistently associated with enhanced antitumor immune activation, increased immune cell infiltration, and improved treatment efficacy. Conversely, antibiotic-induced dysbiosis was linked to reduced pCR rates and poorer clinical outcomes. Microbiome-modulating interventions demonstrated potential in restoring eubiosis and enhancing therapeutic responsiveness. **Conclusions:** Overall, the available evidence supports the microbiome as a promising biomarker and therapeutic target for optimizing treatment strategies and improving outcomes in TNBC.

## 1 Introduction

Triple negative breast cancer (TNBC) is a clinically and biologically aggressive subtype of breast cancer, defined by the lack of estrogen receptor (ER), progesterone receptor (PR), and human epidermal growth factor receptor 2 (HER2) expression [1]. Representing approximately 15–20% of all breast cancer cases, TNBC is associated with high histological grade, rapid disease progression, and poor survival outcomes compared with other molecular subtypes of breast cancer [2]. It is characterized by higher rates of early recurrence and distant metastasis, especially within the first three to five years, with a metastatic spread to the lungs, bones, liver, and central nervous system [3,4].

Because TNBC lacks ER, PR, and HER2 targets, patients with TNBC do not benefit from established endocrine or HER2-targeted drugs, and conventional systemic treatment has relied on chemotherapy in early-stage and metastatic settings. Neoadjuvant chemotherapy is often the standard of care in early-stage disease, with pathological complete response (pCR) a validated prognostic marker [5].

**Figure 1:**
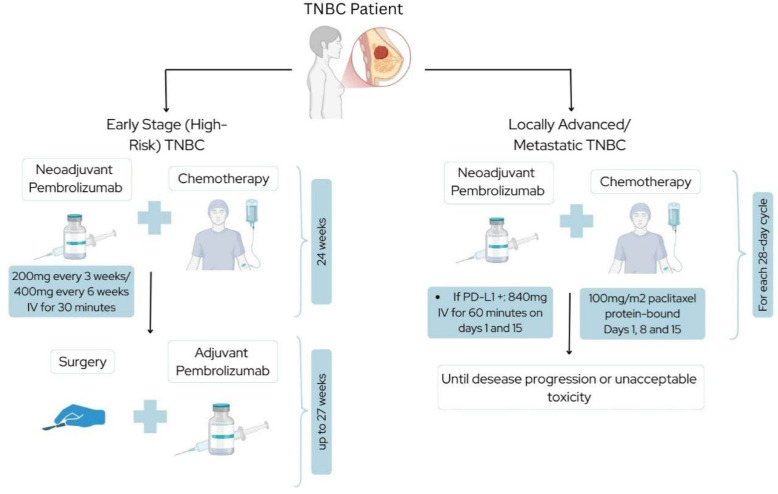
FDA-Approved Therapies for TNBC—schematic representation of therapeutic regimens involving pembrolizumab in patients with triple-negative breast cancer (TNBC) at different disease stages. On the left, treatment for early-stage (high-risk) TNBC includes neoadjuvant pembrolizumab (200 mg every 3 weeks or 400 mg every 6 weeks, IV for 30 min) combined with chemotherapy over 24 weeks, followed by surgery and adjuvant pembrolizumab for up to 27 weeks. On the right, patients with locally advanced or metastatic TNBC receive pembrolizumab (840 mg IV for 60 min on days 1 and 15, if PD-L1 positive) in combination with chemotherapy (protein-bound paclitaxel 100 mg/m^2^ on days 1, 8, and 15 of each 28-day cycle), administered until disease progression or unacceptable toxicity.

However, only a subset of patients achieves a pathological complete response (pCR), defined as the complete absence of residual cancer in the tissue samples removed during surgery after a patient has undergone neoadjuvant systemic therapy, and non-responders face a significantly increased risk of relapse, underscoring the importance of predictive biomarkers and tailored therapy. In recent years, advances in the understanding of TNBC biology have led to the development of novel therapeutic strategies. Therefore, it has been established that combining immunotherapy with conventional chemotherapy is an achievable strategy to improve treatment effectiveness for TNBC (Fig. 1, which schematically represents therapeutic regimens involving pembrolizumab) [6].

Substantial efforts have been directed toward enhancing the effectiveness of both chemotherapy and immunotherapy in TNBC [7,8,9], however, less than 30% of patients with TNBC achieve a pCR, and the incidence and mortality rates remain higher [10,11], highlighting the need for improved prognostic stratification and novel therapeutic strategies.

The normal breast tissue microbiota is composed of a diverse range of commensal and potentially protective bacterial taxa, including *Streptococcus*, *Corynebacterium*, and *Propionibacterium* spp., many of which play important roles in maintaining tissue homeostasis, modulating inflammation, and potentially contributing to mucosal immunity [12,13,14,15]. In the breast cancer context, the microbiome may influence carcinogenesis through multiple mechanisms, including the production of carcinogenic metabolites and the maintenance of pro-inflammatory microenvironments. For instance, certain gut bacteria can enhance immune cell activation, thereby improving response to immunotherapy [16]. In addition, microbial-driven inflammation has been implicated in cancer progression, metastasis, and treatment resistance, as observed with taxa such as *Fusobacterium nucleatum* [17]. In TNBC, the breast microbiome undergoes marked compositional and functional alterations. Studies examining tumor-adjacent and tumor-infiltrating microbiota in TNBC tissues consistently reported reduced microbial diversity, accompanied by enrichment of potentially pathogenic or pro-inflammatory taxa, including *Escherichia coli*, *Fusobacterium nucleatum*, *Enterococcus faecalis*, and members of the *Methylobacteriaceae* family [17,18,19]. Concurrently, a depletion of beneficial commensal bacteria, such as *Shingomonas*, has been observed, which may compromise local immune regulation and tissue integrity [20].

These microbiome alterations are unlikely to be merely passive consequences of tumor development. Accumulating evidence suggests that tumor-associated microbiota may actively shape the tumor microenvironment (TME) by modulating inflammation signalling, immune cell recruitment, angiogenesis, and DNA damage pathways [21,22]. Importantly, breast microbiota dysbiosis in TNBC also carrries significant clinical implications. Altered microbial profiles may directly promote tumor progression by influencing epithelial cell proliferation, survival, and genomic instability. For instance, *E. coli* and *F. nucleatum* can produce genotoxins or activate inflammatory cascades that facilitate tumor growth and immune evasion [16,23,24]. Furthermore, dysbiotic microbiota may affect the efficacy of anticancer therapies, including chemotherapy and immunotherapy. Microbial metabolites and surface molecules, such as lipopolysaccharides, can alter the immune tone of the TME, thereby influencing tumor immunogenicity and responsiveness to immune checkpoint blockade. Conversely, the loss of beneficial microbial taxa may impair antigen presentation, reduce cytotoxic T-cell infiltration, and promote immune escape [16,25,26].

Overall, the interaction between microbiome and TNBC cells, particularly its potential impact on therapeutic response, represents an expanding area of research and highlights the microbiome as a promising target for future personalized treatment strategies in TNBC [27]. This systematic review aims to systematically assess the available evidence on the role of the microbiome in TNBC, with particular emphasis on its impact on therapeutic response and pathological complete response.

## 2 Materials and Methods

This systematic review was conducted and reported in accordance with the Preferred Reporting Items for Systematic Reviews and Meta-Analyses (PRISMA) guidelines (Supplementary Material). The “PICO” framework (P-Population, I-Intervention, C-Comparison, O-Outcome) was used to structure the study and to formulate the guiding research question. Accordingly, the research question addressed in this review was: “Does microbiome influence pathological response in TNBC?”

**(A)**  **Literature Research Strategy**

This systematic review investigated the association between the microbiome and TNBC, with a specific focus on its potential impact and therapeutic relevance in the context of the current standard of care, namely, neoadjuvant chemotherapy.

A rigorous and structured search strategy was implemented to identify relevant studies across two major databases: PubMed and Cochrane, selected to ensure comprehensive coverage of biomedical literature, clinical studies, and reviews. The search strategy incorporated Medical Subject Headings (MeSH) terms: “Triple Negative Breast Neoplasms” (ID: D064726), “Microbiota” (ID: D064307), “Chemotherapy” (ID: D017024), “Pathological Complete Response” (ID: D000095384), and “Immunotherapy” (ID: D007167). Boolean operators (AND) were applied to refine the search and enhance specificity. The literature search was conducted between January and March 2025. Table 1 summarizes the search strategies employed, based on combinations of MeSH terms, to identify the studies included in this review.

**Table 1:** Strategy used for the combination of keywords (Medical Subject Headings terms) to obtain the literature used in this review.

Database	Research Strategy
**PubMed**	Research: “Triple Negative Breast Neoplasms” [MeSH Term] OR “Triple Negative Breast Cancer” AND “Microbiota” AND “Chemotherapy”; “Triple Negative Breast Neoplasms” [MeSH Term] OR “Triple Negative Breast Cancer” AND “Microbiota” AND “Chemotherapy” AND “Pathological Complete Response”; “Triple Negative Breast Neoplasms” [MeSH Term] OR “Triple Negative Breast Cancer” AND “Microbiota” AND “Immunotherapy”; Triple Negative Breast Neoplasms” [MeSH Term] OR “Triple Negative Breast Cancer” AND “Microbiota” AND “Chemotherapy” AND “Immunotherapy”; “Triple Negative Breast Neoplasms” [MeSH Term] OR “Triple Negative Breast Cancer” AND “Microbiota” AND “Chemotherapy” AND “Immunotherapy” AND “Pathological Complete Response” **Filters**: Last 10 years; “English”
**Cochrane**	Research: “Triple Negative Breast Neoplasms [MeSH Term] OR “Triple Negative Breast Cancer” AND “Microbiota” AND “Chemotherapy”; “Triple Negative Breast Neoplasms [MeSH Term] OR “Triple Negative Breast Cancer” AND “Microbiota” AND “Chemotherapy” AND “Pathological Complete Response”; “Triple Negative Breast Neoplasms” [MeSH Term] OR “Triple Negative Breast Cancer” AND “Microbiota” AND “Immunotherapy”; Triple Negative Breast Neoplasms [MeSH Term] OR “Triple Negative Breast Cancer” AND “Microbiota” AND “Chemotherapy” AND “Immunotherapy”; Triple Negative Breast Neoplasms [MeSH Term] OR “Triple Negative Breast Cancer” AND “Microbiota” AND “Chemotherapy” AND “Immunotherapy” AND “Pathological Complete Response” **Filters**: Last 10 years

**(B)**  **Study Selection**

To ensure the quality and relevance of the evidence included in this review, predefined eligibility criteria were applied during the study selection process. This allowed for accurate screening. Study screening was performed independently by two reviewers (Mariana Lopes and Carlos Vila Nova), and any discrepancies were resolved through discussion and consensus.

The inclusion criteria comprised (i) peer-reviewed scientific articles with free full-text availability; (ii) publications written in English; (iii) randomized clinical trials conducted in humans; (iv) non-randomized clinical trials conducted in humans; and (v) studies involving animal models. In addition, only articles published within the last ten years were considered, specifically from December 2015 to December 2024. Studies were excluded if they were: (i) systematic reviews; (ii) case reports or clinical cases; (iii) meta-analysis and (iv) conference abstracts, letters, or commentaries.

**(C)**  **Data Extraction and Analysis**

**Figure 2:**
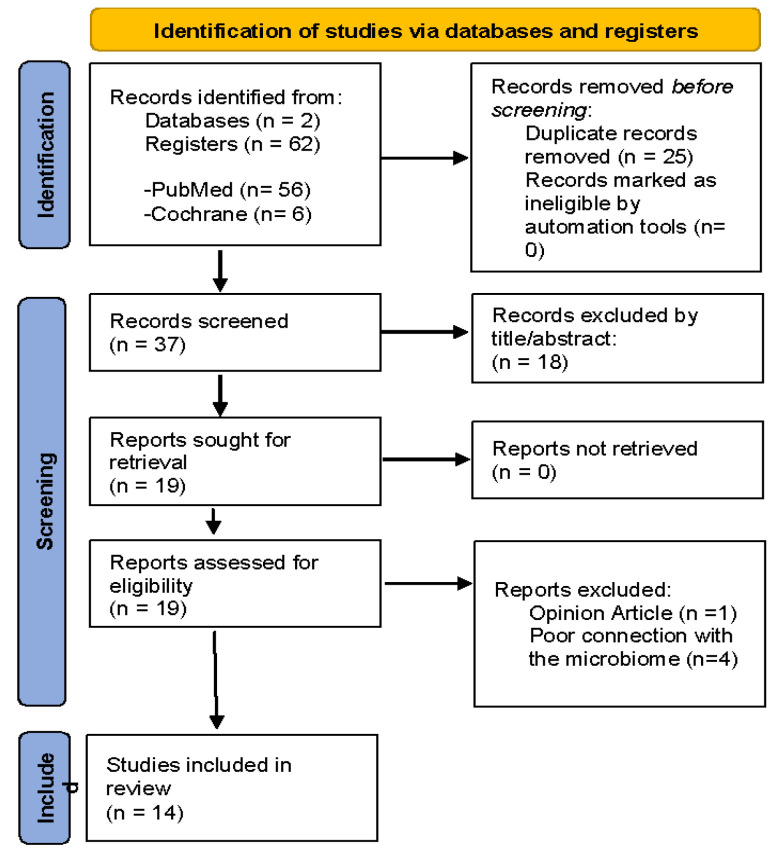
PRISMA Flow Diagram representing each phase of literature research, study selection and final number of studies included in review.

All articles retrieved from the database searches were imported into the PICO Portal platform, a tool widely used in clinical research to support the formulation and management of research questions. The study selection process and methodological approach were conducted in accordance with the PRISMA 2020 guidelines and are summarized in the PRISMA flow diagram presented in Fig. 2. Duplicated articles were eliminated and removed, and the remaining studies were organized to facilitate subsequent screening steps. Titles and abstracts were screened by two independent reviewers (Mariana Lopes and Carlos Vila Nova), based on the predefined inclusion and exclusion criteria, and any discrepancies were resolved through discussion and consensus to determine if they satisfied the previously described criteria and were suitable to be integrated into this systematic review. Studies meeting these criteria were subsequently assessed through full-text review. During full-text evaluation, relevant data were extracted for inclusion in the systematic review.

Based on the database searches, a total of 62 potentially relevant articles were initially identified. Search filters, including publication within the last ten years and English language, were applied; After removal of 25 duplicate records using the PICO platform, 37 articles remained for title and abstract screening.

Following this screening stage, 19 articles were selected for full-text assessment. During full-text review, three articles were excluded: one was classified as an opinion article, and two demonstrated limited relevance to the microbiome, which constituted the central focus of this systematic review. Consequently, 14 studies met the eligibility criteria and were included in the final analysis.

The studies included in this systematic review primarily investigated the influence of the microbiome on the therapeutic response in TNBC. Particular emphasis was placed on the role of microbiota composition and diversity in modulating the efficacy of neoadjuvant chemotherapy (NACT) and immune checkpoint blockade (ICB). The selected articles examine both gut and tumor-associated microbiota, and their potential involvement in shaping the tumor microenvironment, immune activation, and treatment outcomes. Although most studies focused on advanced or high-risk TNBC, both preclinical and clinical investigations evaluating the predictive and prognostic value of the microbiome in neoadjuvant and immunotherapeutic contexts were included.

From each included study, the following data were extracted: (i) publication title; (ii) study design; (iii) study objectives; (iv) study population; (v) interventions assessed; (vi) main outcomes and key findings; (vii) year of publication.

This systematic review was prospectively registered in the Prospective Register of Systematic Reviews (PROSPERO), under the registration number CRD4201079610.

**(D)**  **Study Quality Assessment: *Risk of Bias***

The risk of bias of the studies included in this systematic review was assessed using the Robvis (Risk-Of-Bias VISualization) tool, which provides a standardized and widely adopted framework for evaluating methodological quality and potential sources of bias. The risk of bias was assessed by two independent reviewers (Mariana Lopes and Carlos Vila Nova), and the discrepancies were resolved through discussion and consensus.

**Figure 3:**
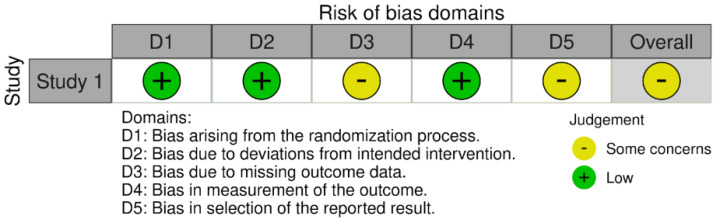
Risk of bias assessment of the included non-randomized studies using the ROBINS-I tool. Risk of bias was evaluated across the following domains: D1: Bias arising from the randomization process; D2: Bias due to deviations from the intended intervention; D3: Bias due to missing outcome data; D4: Bias in measurement of the outcome; D5: Bias in selection of the reported result.

**Figure 4:**
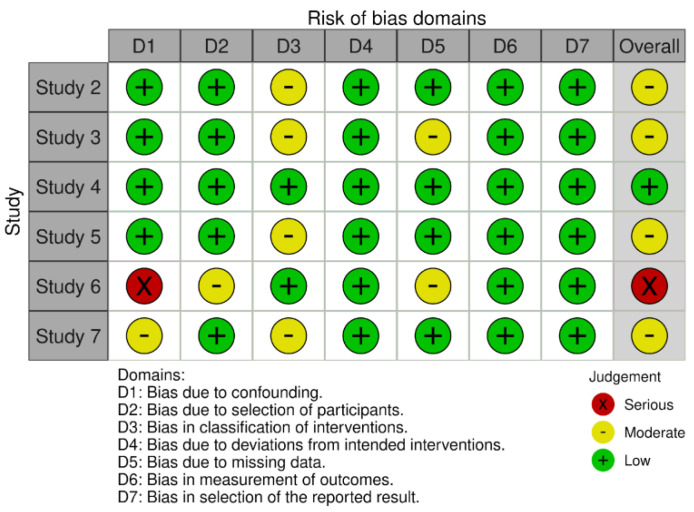
Risk of bias assessment of the included randomized intervention studies using the RoB 2 tool. Risk of bias was evaluated across the following domains: D1: Bias due to confounding; D2: Bias due to selection of participants; D3: Bias in classification of interventions; D4: Bias due to deviations from intended interventions; D5: Bias due to missing data; D6: Bias in measurement of outcomes; D7: Bias in selection of the reported result. Green indicates low risk of bias, yellow moderate risk, orange serious risk, red critical risk, and grey no information.

**Figure 5:**
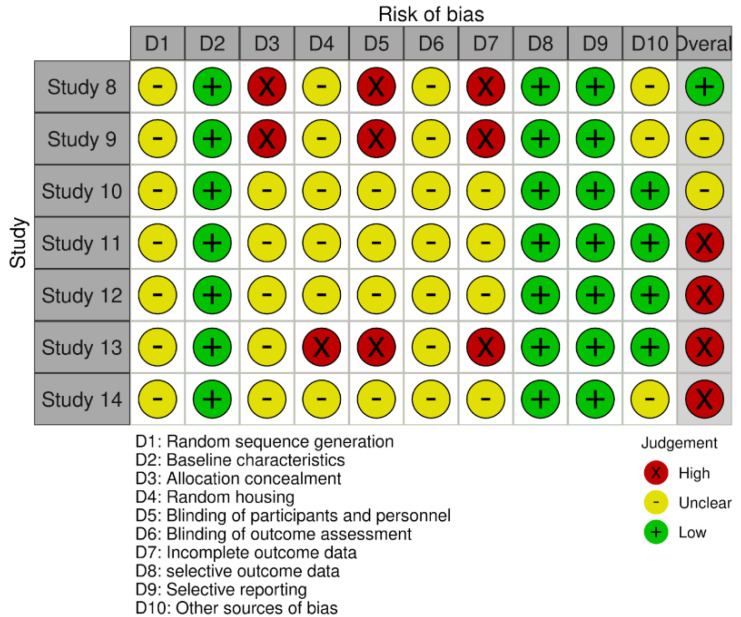
SRYCLE—Murine/animal studies.

Three validated instruments were applied according to the study design. For the non-randomized interventional study (*n* = 1), the ROBINS-I tool was used (Fig. 3). The risk of bias in randomized clinical trials (*n* = 6), was assessed using the revised Cochrane Risk of Bias tool (ROB 2) (Fig. 4). For murine (animal) studies (*n* = 7) the SRYCLE risk of bias tool was applied (Fig. 5).

This assessment was essential to identify potential sources of bias and to support a robust and reliable interpretation of the findings. Risk of bias judgments were summarized using traffic-light plots generated with Robvis and are presented in Fig. 3, Fig. 4 and Fig. 5, providing a concise visual overview of the methodological quality of the included studies.

## 3 Results

Patients with TNBC do not benefit from established endocrine or HER2-targeted treatments due to the absence of the corresponding receptor expression. Consequently, improving survival outcomes and therapeutic response rates requires the exploration of novel treatment strategies, including a combination of chemotherapy with ICB, modulation of the microbiome, and development of vaccine-based approaches.

In this systematic review, fourteen studies, including randomized and non-randomized clinical trials as well as preclinical studies, were identified through database searches and included for analysis within the context of TNBC. Several of the included studies employed human-derived models to evaluate the clinical and biological impact of therapeutic interventions in physiologically relevant settings. Table 2, Table 3, Table 4, Table 5 and Table 6 summarize the methodological characteristics of the studies included.

### 3.1 The MOON Study

The MOON study, as observed in Table 2, was a prospective investigation designed to assess the impact of gut microbiome composition on the likelihood of achieving a pCR in patients with untreated, pathologically confirmed TNBC [28]. A total of 25 patients were enrolled, of whom 56% (*n* = 14), were postmenopausal at diagnosis, and 40% (*n* = 10) were classified as overweight or obese. Regarding disease stage, 88% of the patients (*n* = 22) presented with stage II-III, and 64% (*n* = 16) had nodal involvement in diagnosis.

All the participants were eligible for sequential NACT, consisting of weekly paclitaxel for twelve weeks, followed by four cycles of epirubicin and cyclophosphamide administered every three weeks. Additionally, carboplatin was incorporated into the taxane segment for 92% of the patients (*n* = 23). Five patients (20%) were unable to complete the planned NACT regimens due to treatment-related toxicity. Among those who completed therapy, 56% (*n* = 14) achieved a pCR.

Tumor-infiltrating lymphocytes (TIL) were assessed at two different time points: at diagnosis on core biopsy, where the median TIL level was 30% (range 13.75–46.25%), and after NACT in patients with residual disease (9 out of 11), where the median TIL level as 5% (range 5–15%).

Concerning the gut microbiome analysis, 68 of the 75 collected samples were evaluable. To ensure accurate assessment, the known effects of antibiotic therapy on the gut microbiome were considered, and samples collected within 90 days of antibiotic use were excluded. Taxonomic profiling of the baseline stool samples revealed that the most prevalent *phyla* were *Firmicutes* and *Bacteroidetes*. Patients achieving pCR exhibited significantly higher ∝-diversity, as measured by Shannon diversity index (*p* = 0.049). Although not statistically significant, a trend towards higher richness was observed in pCR patients compared with non-pCR patients (*p* = 0.080 and *p* = 0.162, respectively). Baseline β-diversity analysis showed no significant differences between the pCR and non-pCR groups (*p* = 0.965). Among the main clinicopathological features, a statistically significant difference was observed in body mass index (BMI) between groups (*p* = 0.039); and β-diversity differed significantly between pre- and post-menopausal patients (*p* = 0.035).

Additionally, Bacteroides *eggerthii* demonstrated differential abundance between the two groups (mean relative abundance 0.00% in non pCR vs. and 1.44% in pCR patients). Longitudinal analysis revealed that gut microbiome composition remained stable across timepoints for each patient, in both the pCR and no-pCR groups.

### 3.2 ALICE Trial Phase IIb

In the phase 2b ALICE trial, patients with metastatic or incurable locally advanced TNBC, who had received at most one prior line of chemotherapy for metastatic disease were eligible [29]. The primary objective was to evaluate the gut microbiota composition and its association with clinical outcomes. A total of 70 patients with metastatic TNBC (mTNBC) were enrolled, of whom 28 were randomly assigned to receive chemotherapy alone (placebo-chemo) and 42 to receive chemotherapy combined with atezolizumab (atezo-chemo) (Table 2). The chemotherapy regimen was identical in both arms, consisting of pegylated liposomal doxorubicin (PLD 20 mg/m^2^ intravenously on day 1 of each 14-day cycle) and cyclophosphamide (50 mg orally daily in every 14-day cycle). Atezolizumab (840 mg intravenously) or placebo was administered on day 1 of each cycle in the respective arms.

In the placebo-chemotherapy arm, 82% of patients (*n* = 23) had fecal samples analyzed at baseline. Among those who remained in the study until week 9 (*n* = 22), 82% (*n* = 18) provided paired samples. In the atezolizumab-chemotherapy arm, baseline fecal samples were available for 90% of patients (*n* = 36), and 86% (*n* = 31) of those who continued until week 9 provided paired samples. The median follow-up time was 32.2 months.

No significant differences in α-diversity measures were observed between the two treatment arms. However, in the atezolizumab-chemotherapy arm, patients who provided baseline fecal samples experienced progression-free survival (PFS) [hazard ratio (HR) 0.55; 95% confidence interval (CI) 0.32–0.98]. Clinical benefit, defined as objective response or stable disease at 24-week evaluation, was observed in 56% of patients (*n* = 20/36) in the atezolizumab-chemotherapy arm, compared with 35% (*n* = 8/23) in the placebo-chemotherapy arm. Patients with high baseline Faith’s phylogenetic diversity (PD) exhibited significantly prolonged PFS compared with those with low Faith’s PD (HR 0.56; CI 0.33–0.98; *p* = 0.04). When treatment arms were analysed separately, this association remained in the atezolizumab–chemotherapy arm (HR 0.50; 95% CI 0.21–1.02; *p* = 0.056) but was not observed in the placebo–chemotherapy arm (HR 0.58; 95% CI 0.34–1.86; *p* = 0.57). When Faith’s PD was analyzed as a continuous variable, patients with PFS > 6 months demonstrated significantly higher Faith’s PD compared with those with PFS ≤ 6 months. High baseline Faith’s PD was associated with prolonged PFS in the overall population (HR 0.48; CI 0.27–0.85; *p* = 0.0098). Stratified analyses demonstrated a significant association in the atezolizumab–chemotherapy arm (HR 0.36; 95% CI 0.17–0.77; *p* = 0.0072), but not in the placebo–chemotherapy arm. Importantly, the clinical benefit of atezolizumab was independent of programmed death-ligand 1 (PD-L1) expression, and no association was identified between Faith’s PD and PD-L1 status.

Univariate analysis was conducted to evaluate the association between selected clinical factors, namely older age, fewer than three metastatic sites, and the presence of liver metastasis, and PFS. Older age and fewer than three metastatic sites were significantly associated with improved PFS (*p* = 0.041 and *p* = 0.009, respectively), whereas the presence of liver metastases showed a trend toward poorer PFS (*p* = 0.054) [25]. Bivariate analysis was subsequently performed to adjust for the effect of Faith’s diversity for each of these prognostic factors. The hazard ratios remained largely unchanged after adjustment, indicating that Faith’s PD remained independently and significantly associated with PFS after controlling for these clinical variables.

In the cut-off analysis, univariate modeling demonstrated that Faith’s PD, when analyzed as a continuous variable, was significantly associated with PFS (HR 0.45, *p* = 0.016).

Using the optimized cut-off, patients with high baseline Faith’s PD experience a significant PFS benefit from atezolizumab plus chemotherapy (HR 0.34; CI 0.13–0.86; *p* = 0.018). In contrast, no statistically significant difference in PFS was observed between treatment arms among patients with low Faith’s PD (HR 0.83; 95% CI 0.40–1.74; *p* = 0.62), suggesting that higher baseline Faith’s PD was associated with differential PFS outcomes according to treatment allocation.

Regarding the association between baseline gut microbiota composition and clinical benefit, enrichment of the taxa *Tannerellaceae* and *Enterorhabdus* was observed among patients who achieved clinical benefit. In contrast, patients without clinical benefit exhibited a higher relative abundance of *Bifidobacterium*. Similarly, when treatment arms were analyzed separately, patients in the atezolizumab-chemotherapy arm who derived clinical benefit demonstrated enrichment of *Tannerellaceae*, *Bilophila* and *Enterorhabdus*, whereas those without clinical benefit demonstrated overrepresentation of *Bifidobacterium* and *Anaerostipes*. These patterns were not observed in the placebo-chemotherapy arm.

Despite these trends, only the overrepresentation of *Bifidobacterium* in patients without clinical benefit within the atezolizumab–chemotherapy arm reached statistical significance. Additionally, a high relative abundance of *Bifidobacterium* was associated with significantly lower Faith’s PD compared with patients with low *Bifidobacterium abundance*.

Finally, comparisons of gut microbiota diversity and composition between baseline and fecal samples collected after eight weeks of treatment demonstrated a significant reduction in Faith’s PD over the course of therapy (*p* = 0.0069). When treatment arms were analyzed separately, this reduction reached statistical significance only in the placebo–chemotherapy arm. Regarding treatment-related changes in gut microbiota composition, the relative abundance of *Bifidobacterium* increased significantly after eight weeks of treatment in the atezolizumab–chemotherapy arm, whereas no comparable change was observed in the placebo-chemotherapy arm. Further stratified analysis within the atezolizumab-chemotherapy arm indicated that this increase in *Bifidobacterium* was significant only among patients who achieved clinical benefit.

### 3.3 Tumor Microenvironment Can Predict Chemotherapy Response of Patients with Triple-Negative Breast Cancer Receiving Neoadjuvant Chemotherapy

In the study entitled “*The Tumor Microenvironment Can Predict Chemotherapy Response in Patients with Triple-Negative Breast Cancer Receiving Neoadjuvant Chemotherapy*”, the authors investigated the role of the tumor-associated microbiota in patients with triple-negative breast cancer (TNBC) undergoing neoadjuvant chemotherapy (NACT) [7]. The study also aimed to evaluate whether interactions between the tumor microenvironment and the TNBC-associated microbiota were associated with treatment response over a five-year follow-up period, as observed in Table 2. To this end, breast cancer RNA sequencing data and corresponding clinical characteristics were retrieved from the Gene Expression Omnibus (GEO) and The Cancer Genome Atlas (TCGA), comprising a total of 222 patients.

To characterize overall microbiota composition, the relative abundance of taxa was compared between patients achieving pCR and those without pCR at both the phylum and genus levels. At the phylum level, no statistically significant differences were observed between groups. At the genus level, however, the no-pCR group exhibited a lower overall relative abundance, with *Pasteurella*, *Klebsiella*, and *Vibrio* being particularly prominent. Regarding α-diversity, no significant differences were observed between groups using either Chao 1 or Shannon indices. In contrast, β-diversity differed significantly between the pCR and no-pCR groups (*p* < 0.05), indicating distinct community composition. Differential abundance analyses accounting for potential outliers identified 20 genera with marked differences in relative abundance between groups. At the species level, 24 taxa demonstrated substantial differences in relative abundance. To identify microbial features potentially associated with treatment response, variable importance analysis was performed, using two different types of classifiers: the Support Vector Machine (SVM) and the Random Forest (RF) models. Across these models, *P. pulmonicola*, *Bacillus sonorensis*, *Brucella melitensis* and *Legionella pneumophila* were identified as important discriminative features associated with drug response.

In addition, *Methyloceanibacter* sp., *Kibdelosporangium phytohabitans*, *Plantactinospora* sp. and *Magnetospirillum magneticum* were also identified as important contributors in determining drug responseTo further explore these associations, correlation analysis between the microbiome features and immune cell fraction data was performed and visualized using network plots. These analyses revealed a more complex and interconnected network structure in the pCR group compared with the no-pCR group. In both groups, memory B cells, activated natural killer (NK) cells, activated CD4^+^ memory T cells, and regulatory T cells (Tregs) demonstrated associations with microbiota composition. In the pCR group, taxa such as *Geosporobacter ferrireducens*, *Streptococcus sanguinis*, and *P. pulmonicola* exhibited strong correlations with specific immune cell subsets. Among immune cell populations, resting NK cells, M1 macrophages, and activated CD4^+^ memory T cells showed notable associations with microbial taxa. Conversely, in the no-pCR group, *Plantactinospora* sp. and *Nitrosospira briensis* demonstrated strong correlations with Tregs. Survival analysis further indicated that reduced expression of *Brucella* was associated with improved survival, whereas no significant survival associations were observed for the remaining microbial taxa.

### 3.4 Antimicrobial Exposure Is Associated with Decreased Survival in Triple-Negative Breast Cancer

In the study “Antimicrobial exposure is associated with decreased survival in triple-negative breast cancer”, the authors investigated the association between antimicrobial use during treatment and survival outcomes in patients with TNBC [30]. The final analytic cohort included 772 patients (Table 2), of whom 84% (*n* = 654) received antimicrobials after diagnosis. Among these, 99% (*n* = 649) were treated with antibiotics, 0.8% (*n* = 5) with antifungals, and 24% (*n* = 153) received both antibiotics and antifungals. Among patients who did not receive antimicrobials, 24 (20%) deaths were observed, compared with 153 (23%) deaths among those who used antimicrobials during the study period. The median overall follow-up was 104 months, with a longer median of 121 months for patients who remained alive throughout the observation period.

Using these approaches, any antimicrobial use was not significantly associated with breast cancer-specific survival (BCS) (HR 1.39; CI 0.84–2.32) or overall survival (OS) (HR 1.46; 95% CI 0.93–2.29). In contrast, cumulative total and unique antimicrobial exposures were associated with worse outcomes. Specifically, each additional total monthly prescription was associated with inferior BCS (HR 1.05; 95% CI 1.01–1.08) and OS (HR 1.05; 95% CI 1.02–1.08), while each additional unique monthly prescription was similarly associated with poorer BCS (HR 1.18; 95% CI 1.13–1.24) and OS (HR 1.17; 95% CI 1.12–1.23). Adjustment for potential confounders, including disease severity, did not substantially alter these estimates across any exposure definition: for any antimicrobial exposure, OS HR 1.45 (95% CI 0.93–2.28) and BCS HR 1.40 (95% CI 0.84–2.34); for total exposures, OS HR 1.05 (95% CI 1.02–1.08) and BCS HR 1.05 (95% CI 1.02–1.09); and for unique exposures, OS HR 1.16 (95% CI 1.11–1.21) and BCS HR 1.17 (95% CI 1.12–1.23).

Additionally, the analysis accounted for the potential effects of absolute lymphocyte count (ALC) and absolute neutrophil count (ANC) on survival outcomes. Higher ALC was associated with a reduction in hazard ratios for both OS and BCS; whereas ANC did not appear to influence the HRs for either outcome.

Given the high risk of recurrence in patients with TNBC during the first 2–5 years after diagnosis, the association between antimicrobial use and mortality was evaluated at yearly intervals. A significant and persistent correlation with reduced survival was observed in patients meeting the cumulative antimicrobial exposure criteria during the first three years after diagnosis, with the association diminishing in years four and five.

TIL were also evaluated to assess a potential association with antimicrobial exposure. No association was observed between baseline TIL levels and subsequent antimicrobial use. However, higher continuous TIL scores were significantly associated with achieving a pCR to chemotherapy [median TIL score 3.00 (IQR 1.75–4.25) for pCR vs. 1.00 (0.50–2.00) for non-pCR; *p* = 0.027]. Among these patients, no significant association was found between antimicrobial exposure and pCR status.

### 3.5 Intratumoral Microbiota-Aided Fusion Radiomics Model for Predicting Tumor Response to Neoadjuvant Chemoimmunotherapy in Triple-Negative Breast Cancer

In the study “Intratumoral microbiota-aided fusion radiomics model for predicting tumor response to neoadjuvant chemoimmunotherapy in triple-negative breast cancer”, the authors explored the predictive potential of intratumoral microbiota in early-stage TNBC patients treated with neoadjuvant chemoimmunotherapy (NACI) [31]. By integrating 16S rDNA sequencing, single-cell RNA sequencing and magnetic resonance imaging (MRI)-based radiomics, they developed a non-invasive model to stratify patients according to their likelihood of achieving a pCR [31]. This approach highlights the potential of combining microbiome profiling with imaging-based biomarkers to inform treatment response in TNBC.

A total of 124 female TNBC patients were included (Table 2), with 88 in the training cohort and 36 in the validation cohort. The overall pCR rate was 62.1% (77/12). All patients received standard NACI regimens: nab-paclitaxel (260 mg/m^2^) plus carboplatin (AUC 5) and anti-PD1 (200 mg) every three weeks for four cycles, followed by epirubicin (75 mg/m^2^) plus cyclophosphamide (600 mg/m^2^) with anti-PD1 (200 mg) every three weeks for four cycles. An alternative regimen of nab-paclitaxel (260 mg/m^2^) plus carboplatin (AUC 5) and anti-PD1 (200 mg) every three weeks for 4–6 cycles was also included. All patients underwent pre- and post-NACI breast MRI scans, followed by radical surgery upon completion of therapy.

Intratumoral microbiota load was assessed using PCR, fluorescence *in situ* hybridization (FISH) and immunohistochemistry (IHC) for lipopolysaccharide (LPS), to evaluate bacterial presence within tumor tissues. Tumors that achieved pathological complete response (pCR) exhibited a significantly higher microbial burden compared to non-pCR tumors (*p* < 0.0001).

To explore the underlying mechanisms, single-cell RNA sequencing (scRNA-seq) was performed on samples from six patients. Tumors with high microbial content and pCR demonstrated an increase of FOLR2+ macrophages (46.0% in pCR patients vs. 21.8% in non-pCR patients), a reduction in SPP1+ macrophages (5.76% vs. 22.2%) (*p* < 0.001), associated with poor immunotherapy response and immune suppression. Notably, SPP1+ macrophage abundance was inversely correlated with intratumoral microbiota load. Additionally, pCR patients had a significantly elevated number of IGHM+ plasma cells (53.29%) compared to non-pCR patients (12.14%). In contrast, the non-PCR group presented a higher number of HSP90AA1+ plasma cells (70.14% vs. 16.72%).

### 3.6 A Phase II Clinical Trial of Pembrolizumab and Enobosarm in Patients with Androgen Receptor-Positive Metastatic Triple-Negative Breast Cancer

The phase II clinical trial “*Pembrolizumab and enobosarm in patients with androgen receptor-positive metastatic triple-negative breast cancer*” was an open-label, single-arm study evaluating the safety, efficacy, and biomarker correlates of combining the selective androgen receptor modulator (SARM) enobosarm with the PD-1 inhibitor pembrolizumab in AR+ metastatic TNBC patients [32]. While the primary endpoints focused on objective response rate (ORR) and PFS, the study also explored gut microbiome composition using whole metagenome sequencing of stool samples (*n* = 9) to identify bacterial taxa potentially associated with treatment response and PFS.

The study included 18 patients with histologically confirmed AR+ metastatic TNBC (≥10% nuclear staining by IHC). Patients received pembrolizumab (200 mg IV every three weeks) in combination with enobosarm (18 mg orally daily). PD-L1 status was not used for selection, with 13 of 18 patients (72%) being PD-L1-negative.

Of the 18 patients, 16 were evaluable for response. The clinical benefit rate (CBR) was 25%, and ORR was 12%, including one durable complete response and one partial response. Median PFS was 2.6 months, and median OS was 10.2 months, with one patient remaining in complete remission at 18.9 months of follow-up.

At baseline, overall microbial diversity did not differ significantly between clinical benefit responders (complete response, partial response, or stable disease > 6 months) and non-responders. However, responders exhibited a higher relative abundance of *Bacteroides* and *Alistipes*, whereas non-responders showed greater levels of *Prevotella copri*, *Bacteroides vulgatus* and *Blautia producta*. *E. coli* was more prevalent in non-responders with rapid progression, while the presence of *Alistipes finegoldii* was positively associated with prolonged PFS, although statistical testing was limited by sample size. Moreover, two patients with durable responses (>1-year PFS demonstrated longitudinal stability in their gut microbe, suggesting that microbial composition may predict or contribute to sustained therapeutic benefit. In the following studies, murine models were used to assess the *in vivo* effects of the intervention on tumor progression and immune response under physiologically relevant conditions.

### 3.7 Platycodon Grandiflorum-Derived Extracellular Vesicles Suppress Triple-Negative Breast Cancer Growth by Reversing the Immunosuppressive Tumor Microenvironment and Modulating the Gut Microbiota

In the preclinical study “*Platycodon Grandiflorum-derived extracellular vesicles suppress triple-negative breast cancer growth by reversing the immunosuppressive tumor microenvironment and modulating the gut microbiota*”, Yang et al. (2025) investigated whether *Platycodon grandiflorum*-derived extracellular vesicles (PGEVs) could enhance antitumor efficacy by modulating systemic immunity and the gut microbiome, both as monotherapy and in combination with immune checkpoint blockade (ICB) and chemotherapy [33].

The study, as demonstrated in Table 3, employed orthotopic TNBC murine models of (4T1 tumor-bearing BALB/c mice), with mice randomly assigned to receive vehicle, PGEVs alone, PGEVs plus anti-PD-1 antibody, or PGEVs plus paclitaxel.

Treatment with PGEVs alone significantly suppressed primary tumor growth and pulmonary metastasis compared to control (*p* < 0.001). When combined with anti-PD-1 therapy, PGEVs significantly enhanced immune checkpoint blockade efficacy, resulting in greater tumor reduction and extended survival (*p* < 0.001). Similarly, PGEVs potentiated paclitaxel effects, evidenced by reduced tumor burden and fewer lung nodules (*p* < 0.001).

Mechanistically, the study demonstrated that PGEVs modulated TME byreducing myeloid-derived suppressor cells (MDSCs) and increasing CD8^+^ T cell infiltration within tumor tissue. Flow cytometry and IHC analysis confirmed a marked increase in interferon-γ (IFN-γ)–positive CD8^+^ cytotoxic T lymphocytes (CTLs), alongside a reduction in immunosuppressive mediators, including interleukin-10 (IL-10) and transforming growth factor-β1 (TGF-β1), in PGEV-treated mice. Notably, PGEVs induced a phenotypic shift in tumor-associated macrophages (TAMs) from an M2-like (CD206+) to an M1-like (inducible nitric oxide synthase, iNOS+) profile. Furthermore, a reduction in Treg cells evidenced by decreased Foxp3+ expression, was observed, particularly in combination treatment groups. Collectively, these findings suggest that PGEVs partially reversed TNBC-associated systemic immunosuppression.

Importantly, the study also performed 16S rRNA gene sequencing of fecal samples to evaluate gut microbiome composition. PGEV treatment was associated with a significant increase in ∝-diversity (Shannon index, *p* < 0.05) and principal coordinate analysis revealed distinct clustering of microbial communities in PGEV-treated mice compared with controls. Taxonomic profiling demonstrated an increased relative abundance of potentially beneficial genera including *Lactobacillus* and the phylum *Firmicutes*, while reducing potentially pro-tumorigenic taxa, such as *Bacteroides* and *Proteobacteria*. Correlation analysis suggested positive association between these microbial shifts and enhanced CD8^+^ T cell activity, as well as reduced MDSCs infiltration.

### 3.8 ICB Reprograms Systematic Immune Landscape and TME in Obesity-Associated Breast Cancer

In the study “*ICB reprograms systemic immune landscape and the tumor microenvironment in obesity-associated breast cancer*”, Pingili et al. (2021) conducted a controlled preclinical study to assess the impact of diet-induced obesity on systemic immune profiles, TME features and the therapeutic efficacy of anti-PD-1 ICB in breast cancer [34]. Female C57BL/6J mice were fed either a standard chow diet or a high-fat diet for 12 weeks before orthotopic implantation of E0771 murine breast cancer cells. Following tumor establishment, mice were treated with either anti–PD-1 antibodies or an isotype IgG control [34].

Tumor volume analysis (Table 3) demonstrated that lean mice exhibited a significant response to anti-PD-1 therapy, with reduced tumor growth compared with control-treated animals (*p* < 0.01). This anti-tumor response was associated with increased infiltration of CD8^+^ T cells, and a reduced accumulation of CD11b^+^Gr1^+^ myeloid-derived suppressor cells (MDSCs). In addition, anti–PD-1 treatment promoted a phenotypic shift in tumor-associated macrophages (TAMs) toward a pro-inflammatory profile, characterized by an increase in M1-like TAM and a concomitant decrease in M2-like TAMs, while the overall frequency of F4/80^+^ TAM remained unchanged.

In obese mice, anti-PD-1 treatment resulted in a measurable reduction in tumor burden compared with obese IgG-treated controls; however, the magnitude of tumor suppression was significantly lower than that observed in lean mice receiving anti-PD-1 therapy (*p* < 0.05). Flow cytometry analysis demonstrated increased infiltration of CD8^+^ T cells in tumors from obese mice treated with anti–PD-1. However, the functional cytotoxic response appeared attenuated, as evidenced by reduced levels of IFN-γ–CD8^+^ T cells relative to lean anti–PD-1–treated mice. Similar to findings in lean mice, anti-PD-1 therapy in obese mice also induced a shift in tumor-associated macrophages (TAMs) toward an M1-like phenotype, with a concomitant reduction in M2-like macrophages, while the overall F4/80^+^ TAM population remained unchanged. Despite these immunomodulatory effects, regulatory T cells (Tregs) and myeloid-derived suppressor cells (MDSCs) remained elevated in obese tumors, indicating the persistence of an immunosuppressive tumor microenvironment.

Tumor and systemic cytokine profiling demonstrated that obese mice, irrespective of treatment status, exhibited elevated levels of IL-6, IL-1β and TGF-β, compared with lean controls. These cytokines were increased at baseline and remained persistently elevated after anti-PD-1 therapy.

To evaluate microbial contributions, 16S rRNA gene sequencing was performed on the luminal content from the jejunum (JE, small intestine) and cecum (CE, large intestine) Distinct microbial communities were observed between the two intestinal compartments. Alpha diversity, assessed using Shannon and Simpson indices, was consistently higher in the CE than in the JE. Anti–PD-1 treatment was associated with a reduction in α-diversity in the JE, whereas a reciprocal trend, characterized by a modest increase in α-diversity, was observed in the CE following immunotherapy. β-diversity analyses further demonstrated significant compositional differences between microbial communities across intestinal regions. Separation in microbial β-diversity between tumor-free and tumour-bearing mice was particularly pronounced in CE. Within each intestinal site (JE or CE), tumor-bearing mice exhibited more homogeneous microbial profiles, whereas tumor-free mice showed greater inter-individual variability. Administration of anti-PD-1 therapy was associated with a decrease in ∝-diversity in the JE; conversely, a modest increase in α-diversity was observed in the CE following immunotherapy. Both tumor presence and anti–PD-1 treatment significantly influenced the taxonomic composition of the EC microbiota. Clustering analyses of CE samples indicated that several microbial taxa were more prevalent in tumor-bearing mice than in tumor-free controls. Among these, certain taxa were downregulated following anti–PD-1 therapy, whereas others were selectively enriched relative to IgG2a-treated or tumor-free mice. Taxonomic analysis of the CE in obese mice revealed pronounced changes. In obese tumor-bearing mice treated with IgG2a control, increased relative abundances of the genera *Akkermansia*, *Anaerotruncus*, *Rikenella* and the phylum *AF12* were observed compared with tumor-free counterparts. These taxa were not reduced by anti–PD-1 therapy. Additionally, the presence of tumors was associated with increased levels of *Sutterella*, *unclassified RF39*, *Mucispirillum* and *Bacteroidales*, as well as the families *Enterobacteriaceae* and *Clostridiaceae*; anti–PD-1 treatment partially attenuated these increases. In contrast, tumor-bearing IgG2a treated mice exhibited reduced abundance of *Lactobacillus*, *Bilophila*, *Dorea*, *Ruminococcus*, and members of the *Rikenellaceae* family which were subsequently increased following anti–PD-1 therapy. Furthermore, anti–PD-1 treatment in obese mice was associated with increased relative abundances of *Adlercreutzia*, *Tuberibacter*, *Flexispira*, *Parabacteroides*, *Odoribacter* and the *Helicobacteraceae* family. Anti–PD-1 therapy also increased the abundance of *Bifidobacterium* and *Akkermansia* in the CE of both lean and obese mice. Notably, the effects of anti–PD-1 on several taxa—including *Adlercreutzia*, *Anaerotruncus*, *Ruminococcus*, *Rikenella*, *Mogibacteriaceae* and *Dehalobacterium*—differed between lean and obese mice, indicating a differential microbiota response to immunotherapy across metabolic states.

An additional analysis evaluated the association between tumor volume and selected microbial taxa in obese tumor-bearing mice treated with either IgG2a control or anti-PD-1 therapy. In the JE of obese mice, no significant associations were found. In contrast, a strong positive correlation was observed between tumor size and the relative abundance of the *Enterobacteriaceae* family in the CE of obese tumor-bearing mice (*p* = 0.0002). Anti–PD-1 treatment was associated with a consistent reduction in the relative abundance of *Enterobacteriaceae*.

Lean mice were also included in this analysis and demonstrated a strong negative correlation between tumor volume and the relative abundance of the *Coriobacteriaceae* family, as well as the genera *Bifidobacterium* and *Allobaculum* in the CE. These taxa were not detected in the CE contents of obese mice treated with either IgG2a control or anti-PD-1 therapy but were significantly increased in lean mice receiving anti–PD-1 treatment.

Finally, predicted metabolic pathway analyses of the JE and CE revealed distinct functional profiles between intestinal compartments. Compared with IgG2a-treated controls, anti-PD-1 therapy was associated with alterations in functional pathways predominantly within the EC, where the strongest correlations with tumor volume were observed. Pathways related to ion-coupled transport, DNA replication, oxidative phosphorylation, glycolysis, pyruvate metabolism and arginine and proline metabolism were predicted to be upregulated following anti-PD-1 treatment.

In contrast, pathways involved in amino acid metabolism, methane metabolism, and starch and sucrose metabolism were predicted to be downregulated.

### 3.9 Recombinant-Methioninase-Producing Escherichia Coli Instilled in the Microbiome Inhibits TNBC

The study entitled “Recombinant-methioninase-producing *Escherichia coli* instilled in the microbiome inhibits TNBC” by Kubota et al. (2023) investigated the antitumor effects of recombinant methioninase–producing *E. coli* (JM109-rMETase) in a preclinical model of TNBC [35]. The authors demonstrated that colonization with *E. coli* JM109-rMETase reduced systemic methionine levels and inhibited TNBC tumor growth. Orthotopic TNBC tumors were established by implanting 4T1 cells into the abdominal mammary gland of female athymic nude (nu/nu) mice aged 4–6 weeks. After tumor establishment, as observed in Table 3, a total of 15 mice were randomly allocated into three groups (*n* = 5 per group) and treated by oral gavage twice daily for 14 days. Group 1 received 100 μL of phosphate-buffered saline (PBS) and served as a control. Group 2 received 10^10^ cells/100 μL of non-recombinant *E. coli* JM109 competent cells, serving as an additional bacterial control. Group 3, the treatment group, received 10^10^ cells/100 μL of *E. coli* JM109-rMETase.

Isopropyl-D-thiogalactopyranoside (IPTG) was added to the drinking water in combination with tetracycline (TC) to prevent plasmid loss and to stimulate the production of rMETase in the gastrointestinal tract of the group 3 mice. Tetracycline was also administered to the PBS control group to ensure experimental consistency.

The results showed that, in athymic nude mice (nu/nu), oral administration of *E. coli* JM109-rMETase significantly inhibited the growth of orthotopic 4T1 tumors. Tumor suppression was statistically significant when compared with mice receiving non-recombinant *E. coli* JM109 (*p* = 0.0113) and PBS-treated controls (*p* = 0.0034).

Blood methionine levels were also assessed. Compared with mice receiving non-recombinant *E. coli* JM109 competent cells, mice treated with *E. coli* JM109-rMETase exhibited significantly lower mean blood methionine concentrations on day 15 of treatment. Specifically, mean methionine levels were 85.3 μM in the *E. coli* JM109 competent cell group and 60.5 μM in the *E. coli* JM109–rMETase group, corresponding to an approximate 30% reduction in circulating methionine levels (*p* = 0.0133).

To evaluate treatment tolerability, changes in body weight were monitored throughout the intervention period. Over the 14-day treatment course, no significant differences in body weight were observed in any of the experimental groups, indicating that oral administration of *E. coli* JM109 at a dose of 10^10^ cells/100 μL was well tolerated in athymic nude mice.

### 3.10 Complementarity between Microbiome and Immunity May Account for the Potentiating Effect of Quercetin on the Antitumor Action of Cyclophosphamide in Triple-Negative Breast Cancer Model

In the preclinical study “*Complementarity between microbiome and immunity may account for the potentiating effect of quercetin on the antitumor action of cyclophosphamide in a triple-negative breast cancer model*”, Manni et al. (2023) investigated whether quercetin, a dietary polyphenol with microbiota-modulating properties, could enhance the antitumor efficacy of cyclophosphamide (CTX) in a murine model of TNBC [36].

Female C57BL/6 mice were orthotopically implanted with EO771 TNBC cells and randomly assigned to four treatment groups (Table 3): vehicle control, CTX alone, quercetin alone and quercetin in combination with CTX (Q + CTX). Treatments were administered over 10 days, after which tumor growth, gut microbiota composition and immunological parameters were evaluated.

Tumor volume analysis showed that CTX monotherapy significantly inhibited tumor growth relative to controls. Quercetin alone did not significantly affect tumor size; however, the combination of CTX and quercetin resulted in a further, statistically significant reduction in tumor volume compared with CTX alone, indicating a potential potentiating effect of the polyphenol.

Gut Microbiota composition was analyzed by 16S rRNA gene sequencing fecal samples. α-diversity measured using the Shannon index, was significantly higher in the quercetin-treated groups (quercetin alone and Q + CTX) compared with both CTX-treated and control mice. β-diversity via principal coordinate analysis (PCoA) revealed a distinct clustering of the Q + CTX group, suggesting a unique microbial community structure relative to the other treatment arms. Taxonomic profiling indicated that the Q + CTX group exhibited increased relative abundance of *Lachnospiraceae*, *Akkermansia* and *Ruminococcaceae*, whereas *Bacteroides* and *Muribaculaceae* were reduced. Functional prediction analysis using PICRUSt2 suggested enrichment of pathways involved in short-chain fatty acids (SCFAs) biosynthesis, amino acid metabolism and several biosynthetic processes in the Q + CTX group—in contrast, CTX monotherapy was associated with lower microbial diversity and predicted enrichment of pathways related to nucleotide metabolism and DNA repair.

Immunological profiling of tumor-infiltrating lymphocytes showed that CTX monotherapy increased the presence of CD8^+^ CTL and cells expressing granzyme B and IFN-γ. The combination of Q + CTX further elevated the intratumoral frequencies of both CD8^+^ and CD4^+^ T cells, and increased expression of activation markers such as CD25 and CD69. Additionally, the Q + CTX group exhibited a significant reduction in Treg cells and MDSCs compared with CTX alone. Similar trends were observed systemically, with Q + CTX-treated mice showing higher levels of splenic CD8^+^ T cells and central memory T cells.

Serum cytokine analysis demonstrated elevated levels of IFN-γ and IL-12 levels in the Q + CTX group, alongside decreased concentrations of immunosuppressive cytokines, such as interleukin-10 (IL-10) and transforming growth factor-β (TGF-β), relative to CTX alone. Correspondingly, fecal metabolite quantification showed increased levels of SCFAs, including butyrate and propionate, in mice receiving the Q + CTX combination.

### 3.11 Intestinal Microbiota Influence Doxorubicin Responsiveness in Triple-Negative Breast Cancer

In the preclinical study by Bawaneh et al. (2022), titled “*Intestinal microbiota influenced oxorubicin responsiveness in triple-negative breast cancer*”. The authors investigated the role of gut microbioma in modulating the efficacy of doxorubicin, a standard chemotherapeutic agent for TNBC [37]. Using a syngeneic murine model, female BALB/c mice were orthotopically implanted with 4T1 TNBC cells and stratified into three primary groups: specific pathogen-free (SPF) mice, antibiotic-treated and germ-free (GF) mice. An additional cohort received fecal microbiota transplantation (FMT) following antibiotic-induced microbiota depletion to evaluate microbiota-dependent restoration of chemotherapy response.

The results (Table 3) demonstrated that SPF exhibited a significant reduction in tumor growth following doxorubicin treatment compared to untreated controls (*p* < 0.01). In contrast, antibiotic-treated mice displayed attenuated antitumor responses, with significantly larger tumors than SPF mice (*p* < 0.05), despite receiving identical doxorubicin regimens. Similarly, GF mice showed reduced sensitivity to doxorubicin, highlighting the essential role of the gut microbiota in mediating chemotherapeutic efficacy.

Microbiota depletion was confirmed by 16S rRNA sequencing, demonstrating substantial loss of bacterial richness and diversity after antibiotic treatment. Antibiotic-treated tumor-bearing mice also exhibited reduced intratumoral CD8^+^ cytotoxic T lymphocyte (CTL) infiltration and lower frequencies of granzyme B+ effector T cells relative to SPF controls. Notably, FMT from SPF donors partially restored the antitumor effect of doxorubicin, with tumor volume reductions approaching those observed in SPF mice. This intervention also enhanced CD8^+^ T cell infiltration and increased intratumoral IFN-γ expression. RNA-seq of tumors collected across treatment groups demonstrated that microbiota depletion substantially altered tumor transcriptional profiles. Tumors from antibiotic-treated mice exhibited marked downregulation of interferon-stimulated genes, as well as pathways involved in antigen processing and presentation. In contrast, tumors from SPF mice and those reconstituted by fecal microbiota transplantation (FMT) showed enrichment of immune-related transcriptional signatures, consistent with enhanced antitumor immune activity.

Importantly, no significant differences in systemic toxicity or body weight were observed among the experimental groups, indicating that the observed variations in tumor response were not attributable to differences in overall health status or doxorubicin exposure. Collectively, these findings demonstrate that the presence of a functional gut microbiota enhances TNBC responsiveness to doxorubicin in murine models, an effect associated with increased intratumoral immune activation, particularly augmented CD8^+^ T cell effector function [37].

To further elucidate the impact of microbiota-mediated modulation of treatment response, the subsequent study integrated analyses of human-derived samples with *in vivo* experiments using murine models.

### 3.12 The Microbial Metabolite Trimethylamine N-Oxide Promotes Antitumor Immunity in TNBC

In the study “The microbial metabolite trimethylamine N-oxide promotes antitumor immunity in TNBC” Wang et al. (2022), conducted a comprehensive multi-modal investigation into the role of microbiome and its metabolites in shaping antitumor immunity and ICB response in TNBC [38]. The study integrated multi-omics analysis from a large human TNBC cohort (*n* = 360) with mechanistic validation in enabling the assessment of both correlative and causal relationships between microbiota-derived metabolites and immunotherapy efficacy.

Metagenomic sequencing and untargeted metabolomic profiling of human tumor samples revealed a significant enrichment of tumor-infiltrating *Clostridiales* in the IM TNBC subtype. This microbial signature was associated with elevated levels of trimethylamine N-oxide (TMAO), a metabolite produced by gut and tumor-associated microbiota. Increased intratumoral TMAO levels were observed in immune-active tumors and correlated positively with CD8^+^ T cell infiltration, FN-γ expression, and enhanced ICB-related transcriptional signatures.

To establish causality, *in vivo* experiments were performed in mice bearing orthotopic TNBC tumors. Mice received either systemic or intratumoral TMAO administration in combination with anti-PD-1 therapy. Across both 4T1 and 66cl4 models, as demonstrated in Table 4, the combination treatment significantly suppressed tumor growth compared with monotherapy controls (*p* < 0.01). This antitumor effect was accompanied by increased infiltration of IFN-γ+ CD8^+^ T cells, elevated intratumoral levels of IL-1β and IL-18 and and upregulation of markers associated with GSDME-mediated pyroptosis, an immunogenic form of programmed cell death. Notably, the therapeutic benefit was abolished following CD8^+^ T cell depletion or pharmacological inhibition of the PERK signaling pathway, underscoring the immune-dependent mechanism of action. Translational relevance was further supported by plasma analyses from twelve patients receiving anti-PD-1 therapy, in whom responders exhibited significantly higher circulating TMAO levels compared with non-responders (*p* = 0.002). Elevated plasma TMAO concentrations correlated with prolonged progression-free survival and enhanced *ex vivo* cytotoxic activity of patient-derived CD8^+^ T cells. Additionally, dietary modulation of TMAO levels in murine models, achieved through a choline-enriched diet, significantly increased intratumoral TMAO concentrations and potentiated the efficacy of PD-1 blockade. This effect was reversed by co-treatment with 3,3-dimethyl-1-butanol (DMB), a microbial enzyme inhibitor that suppresses TMAO production. To further dissect the mechanistic underpinnings of these findings, the study integrated *in vitro* assays using TNBC cell lines with *in vivo* validation in murine models, providing robust evidence for a microbiota–metabolite–immune axis in modulating immunotherapy response in TNBC.

### 3.13 Detoxified Pneumolysin Derivative A146Ply Inhibits Triple-Negative Breast Cancer Metastasis Mainly via Mannose Receptor-Mediated Autophagy Inhibition

In the preclinical study “*Detoxified pneumolysin derivative A146Ply inhibits triple negative breast cancer metastasis mainly via mannose receptor-mediated autophagy inhibition*”, Zhang et al. (2024) evaluated the therapeutic potential of a detoxified pneumolysin derivative, ΔA146Ply, in TNBC using both *in vitro* human cell lines and *in vivo* murine models [39]. The study aimed to determine whether ΔA146Ply could supress tumor growth and metastasis and to elucidate the underlying mechanisms, with a particular focus on autophagy regulation and innate immune signaling. In an orthotopic EO771-LMB TNBC mouse model, intravenous administration of ΔA146Ply (25 μg per mouse every three days for 21 days), as observed in Table 5, resulted in a significant reduction in primary tumor volume compared with vehicle-treated controls (*p* < 0.01). In addition, pulmonary metastatic burden was markedly reduced in the ΔA146Ply-treated mice, as demonstrated by both macroscopic nodule count and histopathological analysis using H&E staining, suggesting robust anti-metastatic.

Mechanistic analyses revealed that ΔA146Ply preferentially localized to tumor-associated macrophages (TAMs), particularly CD206^+^ mannose receptor–expressing cells, consistent with selective targeting of M2-like macrophage populations. Confocal microscopy and flow cytometry confirmed enhanced uptake of ΔA146Ply by CD106+ TAM, leading to inhibition of autophagic flux, as evidenced by reduced LC3-II expression and accumulation of p62/SQSTM1. These effects were not observed in CD206—populations, supporting receptor-specific activity.

Transcriptomic profiling of tumor tissues using RNA sequencing, followed by gene set enrichment analysis (GSEA), demonstrated downregulation of autophagy-related genes and suppression of pathways involved in tumor invasiveness and extracellular matrix remodeling. Moreover, ΔA146Ply treatment induced upregulation of interferon-related signaling pathways, including IFN-γ–associated gene networks, although immune checkpoint blockade interventions were not directly evaluated in this study.

While the gut microbiota was not directly assessed, ΔA146Ply derived from *Streptococcus pneumoniae* retains immunomodulatory properties of microbial origin. The observed antitumor effects were linked to modulation of innate immune receptors and macrophage polarization within the tumor microenvironment.

Collectively, these findings indicate that ΔA146Ply suppresses TNBC growth and metastasis *in vivo* by inhibiting autophagy in CD206^+^ TAMs and enhancing antitumor immune signaling. This study highlights the therapeutic potential of microbial-derived agents to modulate innate immune pathways and reshape the tumor immune microenvironment in TNBC.

To further investigate the mechanistic interplay between microbial signals and immunotherapy responsiveness, the subsequent study employed an *ex vivo* organoid co-culture model to evaluate the impact of microbiota-derived factors on immune-mediated responses to checkpoint inhibition.

### 3.14 Immuno-Reactive Cancer Organoid Model to Assess Effects of the Microbiome on Cancer Immunotherapy

In the study “*Immuno-reactive cancer organoid model to assess effects of the microbiome on cancer immunotherapy*”, Shelkey et al. (2022) developed an *ex vivo* co-culture platform comprising murine-derived breast cancer organoids generated from MMTV-PyMT tumors and matched autologous splenocytes [40]. This model was designed to evaluate immunomodulatory effects of microbiota-derived soluble factors on responses to anti–PD-1 ICB. To recapitulate microbiome influence, organoid-immune cell co-cultures were exposed to sterile fecal filtrates (FF) obtained from either antibiotic-treated or untreated (microbiota-intact) mice. As demonstrated in Table 6, the experimental design included four treatment conditions: untreated control, anti–PD-1 antibody alone, FF alone, and combined anti–PD-1 plus FF. Cultures were maintained for five days, after which multiple immunological and tumor response endpoints were assessed. Exposure to FF from microbiota-intact mice resulted in significantly enhanced immune activation compared with FF derived from antibiotic-treated mice. Notably, co-treatment with anti–PD-1 and FF from untreated mice significantly increased the proportion of CD8^+^ T cells among TILs (*p* < 0.01), accompanied by elevated expression of granzyme B and IFN-γ, as determined by flow cytometry. In contrast, these cytotoxic and inflammatory markers were markedly reduced in co-cultures treated with FF from antibiotic-treated mice, indicating the loss of immune-potentiating microbial signals. Consistent with enhanced CTL activation, co-treatment with anti-PD-1 and FF from microbiota-intact mice led to increased caspase-3 expression within organoid tumor cells, reflecting higher apoptosis. Organoids treated with anti-PD-1 in combination with antibiotic-derived FF did not exhibit significant increases in apoptotic markers compared to control conditions, further supporting the dependence of immune-mediated tumor killing on microbiota-derived factors.

Organoid viability was significantly reduced only in the combined anti–PD-1 and microbiota-intact FF condition, while remaining unchanged in cultures exposed to FF from antibiotic-treated mice. These findings were supported by immunofluorescence microscopy and qPCR analysis, which confirmed upregulation of inflammatory gene expression and apoptosis-related pathways in the microbiota-intact treatment group.

Importantly, the use of sterile fecal filtrates ensured that the observed effects were mediated exclusively by soluble microbial metabolites or structural components rather than by live bacterial colonization. This *ex vivo* organoid-based system therefore provides a controlled and physiologically relevant platform to dissect microbiome-derived immunomodulatory signals within a defined tumor microenvironment.

**Table 2:** Clinical and translational human studies on the microbiome’s influence in TNBC and treatment response to chemotherapy and immunotherapy.

Title	Year	Study Type	Objective	Population	Treatment Regimen	Key Results	Reference
**Characterization of Gut Microbiome Composition in Patients with TNBC Treated with NACT**	2023	Observational	To assess gut microbiome feasibility, its link to treatment response, and changes during neoadjuvant chemotherapy in early TNBC	25 TNBC vs. 25 healthy controls	No treatment	↓ Diversity (*p* < 0.05); ↑ Escherichia-Shigella, Proteobacteria. ↓ Faecalibacterium, Firmicutes; E. Shigella linked to ↑ IL-6 & TNF-α	[28]
**Gut Microbiota Diversity is Prognostic and Associated with Benefit from Chemoimmunotherapy in mTNBC**	2024	Clinical Trial (Phase IIb)	To explore gut microbiota associations with response and toxicity to chemo ± PD-L1 blockade, and its changes over time during treatment	70 mTNBC patients	Atezolizumab + PLD + low-dose cyclophosphamide vs. placebo	High Faith’s PD → longer PFS (HR = 0.34, *p* = 0.018); ↓ diversity during treatment; *Bifidobacterium* enriched in non-responders	[29]
**TME Can Predict Chemotherapy Response of Patients with TNBC Receiving NAC**	2023	Bioinformatic	To identify biomarkers and develop a tumor microbiome-based model to predict response to NACT in TNBC	88 TNBC (GEO), 115 (TCGA)	Neoadjuvant chemo (anthracycline, taxane, cyclophosphamide)	pCR vs. no-pCR showed different β-diversity (*p* < 0.05); ↑ *Pandoraea*, *Brucella* in pCR; ML model predicted response (AUC > 0.80)	[7]
**Antimicrobial Exposure is Associated with Decreased Survival in TNBC**	2023	Retrospective Cohort	To assess the impact of antimicrobial exposure on survival and lymphocyte levels in TNBC patients treated without ICB	772 Stage I–III TNBC	Standard chemo (non-ICB)	Each added antimicrobial/month → ↑ risk of death (OS HR = 1.17, BCS HR = 1.18); effect independent of stage, ANC	[30]
**Phase II Clinical Trial of Pembrolizumab and Enobosarm in Patients with AR^+^ mTNBC**	2021	Clinical Trial (Phase II, open-label, single arm)	To assess safety, tolerability, and clinical efficacy of pembrolizumab + enobosarm, and explore associated immune and microbiome biomarkers	18 AR+ mTNBC patients	Pembrolizumab 200 mg IV q3w + Enobosarm 18 mg orally daily	CBR: 25%; RR: 12%; PFS: 2.6 months. Well-tolerated. Suggests modest benefit and potential synergy in AR+ tumors despite lack of PD-L1 preselection. Microbiome analyzed, but no major associations reported	[32]
**Intratumoral microbiota-aided fusion radiomics model for predicting tumor response to NACI in TNBC**	2025	Prospective translational study	To develop and validate a noninvasive model combining MRI radiomics and intratumoral microbiota to predict pCR to NACI in TNBC	124 early-stage TNBC patients	NACI with: nab-paclitaxel (260 mg/m^2^, d1) + carboplatin (AUC 5, d1) + anti–PD-1 (200 mg, d1) q3w × 4 cycles ± epirubicin (75 mg/m^2^, d1) + cyclophosphamide (600 mg/m^2^, d1) q3w × 4 cycles	↑ Microbiota load in pCR tumors; ↓ SPP1^+^ macrophages; fusion MRI–microbiota model predicted pCR (AUC = 0.945 train, 0.873 validate); balanced sensitivity (77.3%) & specificity (78.6%)	[31]

Abbreviations are as follows: Triple Negative Breast Cancer (TNBC), Neoadjuvant Chemoimmunotherapy (NACI), Interleukin (IL), Tumor Necrosis Factor (TNF), Metastatic Triple Negative Breast Cancer (mTNBC), Phylogenetic diversity (PD), Progression-Free Survival (PFS), Hazard Ratio (HR), Tumor Microenvironment (TME), Neoadjuvant Chemotherapy (NACT), Gene Expression Omnibus (GEO), The Cancer Genome Atlas (TCGA), Pathological Complete Response (pCR), Machine Learning (ML), Area Under the Curve (AUC), Overall Survival (OS), Breast cancer-specific Survival (BCS), Absolute Neutrophil Count (ANC), Androgen-Receptor Positive (AR+), Intravenous (IV), Once Every Three Weeks (q3w), Clinical Benefit Rate (CBR), Response Rate (RR), Magnetic Resonance Imaging (MRI).

**Table 3:** Clinical and translational murine/mice studies on the microbiome’s influence in TNBC and treatment response to chemotherapy and immunotherapy.

Title	Year	Study Type	Objective	Population	Treatment Regimen	Key Results	Reference
**PGEVs Suppress TNBC Growth by Reversing the Immunosuppressive TME and Modulating the GM**	2022	Preclinical *in vivo* murine	To assess whether PGEVs suppress TNBC progression by modulating the tumor immune microenvironment and GM, and enhancing response to ICB and NACT	Female BALB/c mice (6–8 weeks), orthotopically implanted with 4T1 TNBC cells	- PGEVs (oral gavage) - PGEVs + anti–PD-1 antibody - PGEVs + paclitaxel - Control (vehicle)	PGEVs induced ↑ ROS, apoptosis, M1 TAMs polarization, ↑ pro-inflammatory cytokines. Modulated GM; ↓ immunosuppressive TME; enhanced anti-tumor immunity and suppressed tumor growth	[33]
**ICB Reprograms Systematic Immune Landscape and TME in Obesity-Associated Breast Cancer**	2021	Preclinical *in vivo* murine	To investigate how obesity alters systemic immunity and affects the response to anti–PD-1 therapy in breast cancer, including changes to the GM and TME	Female C57BL/6J mice, diet-induced obese and lean controls, implanted orthotopically with E0771 breast cancer cells	Anti–PD-1 antibody (intraperitoneal injection) vs. IgG control; animals stratified by lean vs. obese status	Anti-PD-1 reversed obesity-induced immunosuppression, ↓ tumor burden. Identified microbial signature predictive of ICB efficacy. Microbiota linked to differential immune responses and metabolic pathways	[34]
**Recombinant-methioninase-producing *E. coli* Instilled in the Microbiome Inhibits TNBC in an Orthotopic Cell-line Mouse Model**	2023	Preclinical *in vivo* mice	To evaluate whether oral administration of recombinant *E. coli* JM109 rMETase can reduce plasma methionine levels and inhibit tumor growth in a TNBC mouse model	Female BALB/c nude mice orthotopically implanted with 4T1 TNBC cells	Oral gavage with recombinant *E. coli* JM109 expressing rMETase vs. control (PBS or non-recombinant bacteria)	Gut-colonizing *E. coli* JM109-rMETase reduced blood methionine levels (–30%) and significantly suppressed tumor growth compared to controls, suggesting microbiome-delivered metabolic therapy potential.	[35]
**Complementarity Between Microbiome and Immunity May Account for the Potentiating Effect of Quercetin on the Antitumor Action of Cyclophosphamide in a TNBC Model**	2023	Preclinical *in vivo* mice	To evaluate whether quercetin enhances the antitumor efficacy of cyclophosphamide via microbiome and immune modulation in TNBC	Female C57BL/6 mice orthotopically implanted with EO771 TNBC cells	Oral quercetin (60 mg/kg/day), intraperitoneal cyclophosphamide (100 mg/kg every 5 days), alone or in combination, over a 10-day period	Quercetin enriched microbiota (↑Akkermansia), modulated β-diversity (*p* < 0.001). Quercetin + Cyclo ↓ tumor growth (*p* < 0.05), ↑ CD3^+^, CD4^+^, NK cells; ↓ Tregs. Complementary microbiome & immune mechanisms explained synergy	[36]
**Intestinal Microbiota Influence Doxorubicin Responsiveness in Triple-Negative Breast Cancer**	2022	Preclinical *in vivo* mice	To determine whether the gut microbiota influences the antitumor response to doxorubicin in TNBC	Female BALB/c mice orthotopically injected with 4T1 TNBC cells; included SPF, antibiotic-treated, and germ-free groups	Intraperitoneal doxorubicin (5 mg/kg) administered every 3 days for 2 weeks; with or without prior antibiotic treatment or fecal microbiota transplantation (FMT)	Doxorubicin responders had increased *A. muciniphila*, *R. intestinalis*, *C. clostridioforme*, *O. ruminantium*. FMT from high-fat diet mice reduced response; dysbiosis linked to reduced efficacy and increased metastasis.	[37]

Abbreviations are as followed: Platycodon Grandiflorum-Derived Extracellular Vesicles (PGEVs), Triple-Negative Breast Cancer (TNBC), Tumor Microenvironment (TME), Gut Microbiota (GM), Reactive Oxygen Species (ROS), Tumor-Associated Macrophages (TAMs), Immune Checkpoint Blockade (ICB), *Escherichia coli* (*E. coli*), Recombinant-Methioninase (rMETase), Phosphate-Buffered Saline (PBS), Fecal Microbiota Transplantation (FMT).

**Table 4:** Clinical and translational human and murine study on the microbiome’s immunomodulatory role in TNBC and implications for treatment with immunotherapy.

Title	Year	Study Type	Objective	Population	Treatment Regimen	Key Results	Reference
**The Microbial Metabolite TMAO Promotes Antitumor Immunity in TNBC**	2022	Translational study	To evaluate the effect of microbiota-derived TMAO on antitumor immunity and response to anti–PD-1 therapy	- 360 TNBC patients (FUSCCTNBC cohort) - BALB/c mice with orthotopic 4T1 or 66cl4 TNBC tumors	TMAO (systemic/intratumoral); Anti–PD-1 therapy; CD8^+^ T cell depletion; Choline diet ± DMB	High TMAO → ↑ CD8^+^ T cells, ↑ IFN-γ, ↓ tumor growth. TMAO induced GSDME-mediated pyroptosis via PERK. Synergized with anti-PD-1. TMAO linked to Clostridiales; predicted immunotherapy response	[38]

Abbreviations are as follows: Trimethylamine N-Oxide (TMAO), Triple-Negative Breast Cancer (TNBC), 3,3-Dimethyl-1-Butanol (DMB), Interferon-gama (IFN-γ).

**Table 5:** Clinical and translational murine/mice + cell line study on the microbiome’s influence in TNBC and treatment response to chemotherapy and immunotherapy.

Title	Year	Study Type	Objective	Population	Treatment Regimen	Key Results	Reference
**Detoxified pneumolysin derivative A146Ply inhibits TNBC metastasis mainly via mannose receptor-mediated autophagy inhibition**	2024	Preclinical *in vivo* and *in vitro*	To investigate whether the detoxified pneumolysin derivative ΔA146Ply inhibits TNBC metastasis and to elucidate the mechanism of action via mannose receptor–mediated autophagy inhibition	*In vitro*: human TNBC cell lines (e.g., MDA-MB-231); *In vivo*: female C57BL/6J mice with EO771-LMB orthotopic and metastatic TNBC models	Intravenous administration of ΔA146Ply (25 μg/mouse every 3 days for 21 days); vehicle and positive controls used for comparison	ΔA146Ply inhibited metastasis via mannose receptor–mediated autophagy and TGF-β1 suppression; combined with doxorubicin it enhanced survival and tumor inhibition	[39]

Abbreviations are as follows: Triple-Negative Breast Cancer (TNBC).

**Table 6:** Clinical and translational organoid study on the microbiome’s influence in TNBC and treatment response to chemotherapy and immunotherapy.

Title	Year	Study Type	Objective	Population	Treatment Regimen	Key Results	Reference
**Immuno-reactive cancer organoid model to assess effects of the microbiome on cancer immunotherapy**	2022	Preclinical *ex vivo* (organoid model)	To develop an immuno-reactive organoid co-culture system to evaluate the impact of microbiome-derived factors on tumor immune responses and checkpoint inhibitor efficacy	Mouse-derived breast cancer organoids (MMTV-PyMT), co-cultured with autologous splenocytes; microbial stimuli derived from fecal filtrates (Abx-treated vs. untreated mice)	Organoids were treated with anti–PD-1 antibodies (10 μg/mL) with or without fecal filtrate treatment (from antibiotic-treated or untreated mice) for 5 days	Bacterial metabolites (e.g., HIP, SB, IN) increased CTLs viability and apoptosis; combination therapy enhanced granzyme B, cleaved caspase-3, and CD8 expression.	[40]

## 4 Discussion

TNBC remains one of the most aggressive and therapeutically challenging subtypes of breast cancer Although TNBC often exhibits initial sensitivity to chemotherapy, particularly in the neoadjuvant setting (NACT), recurrence rates remain high and long-term survival outcomes are poor, especially among patients who fail to achieve a pathological complete response (pCR) [41]. Recently, increasing attention has been directed toward the microbiome, including both gut and tumor-associated microbial communities, as a potential modulator of host immune tone, systemic inflammation, antigen presentation and responsiveness to anticancer therapies [42].

**Figure 6:**
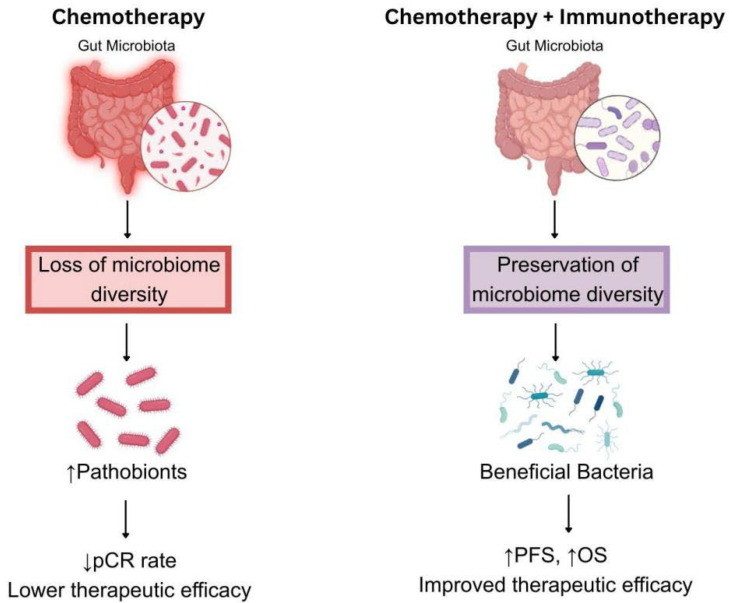
Chemotherapy alone tends to destroy microbial diversity, promoting dysbiosis and a worse therapeutic response. On the other hand, chemotherapy plus immunotherapy preserves or restores diversity, allowing the enrichment of beneficial bacteria that reinforce antitumor efficacy and reduce resistance. Image provided by Servier Medical Art (https://smart.servier.com), licensed under CC BY 4.0 (https://creativecommons.org/licenses/by/4.0/).

Within this context, integrated therapeutic strategies combining chemotherapy, immune checkpoint blockade (ICB), and modulation of host–microbiome–immune interactions have been explored to improve treatment efficacy. Emerging evidence suggests that the composition and diversity of the gut and tumor-associated microbiome may significantly influence treatment outcomes by shaping systemic immune responses and modulating sensitivity to both chemotherapy and immunotherapy. In this review, comparisons between chemotherapy alone and chemotherapy combined with immunotherapy highlight the potential for microbial communities to differentially interact with these treatment modalities, thereby influencing therapeutic efficacy (Fig. 6, which schematically illustrates the impact of chemotherapy versus combined chemoimmunotherapy on the gut microbiota).

The following studies, conducted in human-derived models, provide translationally relevant insights by capturing patient-specific responses to therapy within clinically meaningful settings. Vernaci et al. (2023) performed gene sequencing–based longitudinal profiling of the gut microbiome in patients with early-stage TNBC undergoing NACT to assess its association with treatment response [28]. The study demonstrated a significant association between baseline gut microbiome α-diversity and achievement of pCR, with patients attaining pCR exhibiting significantly higher baseline α-diversity compared with those with residual disease (*p* = 0.049). A trend toward increased microbial richness and evenness was also observed in patients achieving pCR, although this did not reach statistical significance (*p* = 0.162). These findings are consistent with observations in other malignancies, including melanoma, non-small cell lung cancer, and hepatocellular carcinoma, where higher gut microbial diversity has been associated with improved responses to both chemotherapy and ICB [28].

No significant differences in β-diversity were detected between pCR and non-pCR groups, suggesting that broad compositional shifts in the microbiome may not be required to influence therapeutic response. The authors acknowledged the limited sample size as a key limitation, which may have reduced the power to detect more subtle or taxon-specific compositional changes [28].

At the species level, the study identified a differential abundance of *Bacteroides eggerthii* in patients who achieved pCR (1.44%) compared to those who did not (0.00%) [28]. In pre-clinical models, this bacterial species has been associated with anti-inflammatory and anticarcinogenic effects, partly mediated through the metabolism of dietary flavonoids, suggesting a potential mechanism by which it could influence chemotherapeutic response in TNBC. Notably, *Bacteroides eggerthii* is capable of metabolizing quercetin into bioactive derivatives, including 2,4,6-trihydroxybenzoic acid and 3,4-dihydroxybenzoic acid, metabolites shown to exert antiproliferative effects and modulate NF-kB signaling pathways [43,44,45].

This study further evaluated longitudinal microbiome dynamics at three times: before chemotherapy (t0), one-week after treatment initiation (t1) and eight weeks post-treatment (t2). After exclusion of samples affected by antibiotic exposure, longitudinal analysis of a patient subset revealed no substantial changes in overall microbial composition across timepoints. Specifically, the *Firmicutes*/*Bacteroidetes* ratio and global taxonomic distribution remained largely stable throughout the treatment course [28].

Importantly, patients achieving pCR also exhibited higher levels of stromal TILs, a well-established prognostic and predictive biomarker in TNBC. Although the study did not directly correlate specific microbial taxa with TIL density, on current presence of elevated microbial α-diversity and increased stromal TILs suggests a potential biological synergy between the gut microbiome and antitumor immune activation [28]. This hypothesis is supported by existing evidence indicating that commensal microbes can enhance MHC-I and MHC-II expression on antigen-presenting cells, thereby improving the priming of TIL precursors in lymphoid tissues. Additionally, microbiota-regulated cytokine and chemokine profiles facilitate the homing of effector T cells into the TME [46,47,48].

The observed longitudinal stability of the gut microbiota suggests that chemotherapy alone may not drastically disrupt the gut microbiota ecosystem, especially in patients without recent antibiotic exposure [49]. This finding underscores the potential clinical relevance of baseline microbiome profiling as a relatively durable biomarker for predicting NACT efficacy in TNBC. Moreover, the relative stability of microbial composition throughout treatment highlights a potential therapeutic opportunity for pre-treatment microbiome modulation aimed at enhancing treatment responsiveness [28].

Despite these promising findings, Vernaci et al. acknowledged several limitations, including a small sample size (*n* = 25), the absence of functional microbiome profiling, due to reliance on 16S rRNA gene sequencing and the absence of dietary and lifestyle data—factors known to influence gut microbiome composition and function [28].

Similarly, the ALICE trial, reported by Ullern et al. (2025) demonstrated that baseline gut microbiota α-diversity, measured via Faith’s PD, was significantly associated with prolonged PFS. This association was observed in the overall study population but was particularly pronounced in patients receiving atezolizumab plus chemotherapy, and not in those treated with chemotherapy alone. These findings suggest that α-diversity may serve as a predictive biomarker of immunotherapy benefit rather than a general prognostic indicator [29].

Notably, a significant decline in Faith’s PD was observed in both arms, indicating that chemoimmunotherapy may disrupt microbial homeostasis. Such reductions may reflect chemotherapy-induced mucosal damage, which can compromise microbial niches [29].

This study further reported that specific bacterial taxa were differentially enriched according to treatment response. Genera, such as *Tannerellaceae* and *Enterorhabdus*, were more abundant in responders, whereas *Bifidobacterium* was significantly overrepresented in non-responders within the atezolizumab arm. This finding contrasts with prior evidence linking *Bifidobacterium* to enhanced immune checkpoint blockade (ICB) responses in melanoma and lung cancer models [29,50,51], underscoring the context-dependent nature of microbiota-immune interactions among different cancer types and treatment approaches.

Although tumor-infiltrating TIL was not directly assessed, the observed association between microbial diversity and immunotherapy response suggests a potential indirect modulation of TIL presence and function within TME. This association is supported by preclinical and translational studies demonstrating that microbiota-derived SCFA-, such as butyrate, can promote epigenetic programming of effector T cells, promoting memory formation and cytotoxic activity. In addition, specific taxa, including *Akkermansia muciniphila*, and *Faecalibacterium prausnitzii*, have been shown to facilitate the recruitment of antigen-specific lymphocytes into tumors [52,53,54]. Collectively, these findings suggest that microbiota-driven enhancement of TIL recruitment may represent a key intermediary mechanism linking gut microbial diversity to improved immunotherapy efficacy.

Importantly, multivariate analyses demonstrated that Faith’s PD remained significantly associated with treatment response after adjustment for established prognostic factors, including age, number of metastatic sites, and liver involvement, supporting its potential role as an independent biomarker. Nevertheless, the authors acknowledged several limitations, including possible confounders such as antibiotic exposure, diet, comorbidities, host genetics and modest sample size [29].

Kim et al. (2024) integrated bulk RNA-sequencing-derived microbiome profiling with immune cell deconvolution to investigate tumor-local microbiota-immune interactions and their association with treatment response and survival in TNBC patients undergoing NACT [7]. The authors reported significant differences in microbial β-diversity between patients achieving pCR and those with residual disease (non-pCR), indicating response-associated shifts at the community level. In contrast, no significant differences in α-diversity were observed, suggesting that microbial composition and structure potentially influence therapeutic outcomes in a therapeutic context [7].

At the genus and species levels, distinct microbial taxa were enriched in either the pCR or non-pCR groups. Correlations between microbial taxa and TIL were explored using co-occurrence network analysis, revealing that patients achieving pCR exhibited more complex and interconnected microbiome-immune interaction networks compared with non-responders. *Brucella melitensis* and *Pandoraea pulmonicola* emerged as key taxa associated with the prediction g of treatment response [7]. Furthermore, pCR was characterized by co-associations between taxa such as *Geosporobacter ferrireducens* and *Streptococcus sanguinis* and higher levels of resting NK cells, whereas the non-pCR group was enriched for *Nitrosospira briensis* and *Plantactinospora* sp. In conjunction with FOXP3+ regulatory T cells, a population known to suppress antitumor immune responses [7].

Collectively, these findings suggested that tumor-associated microbial communities may modulate the immunological landscape of the TME, influencing the balance between effector and immunosuppressive immune states. Enrichment of Treg cells is commonly associated with immune evasion and chemoresistance, whereas increased M1 macrophage polarization and NK cell activity have been linked to efficient tumor clearance. Furthermore, survival analysis demonstrated that immune profiles associated with specific microbial signatures correlated clinical outcomes, as lower M2 macrophage abundance was significantly associated with improved 5-year survival (*p* = 0.0097). In addition, trends toward improved prognosis were observed in patients with higher levels of resting NK cells and M1 macrophages, supporting their protective role in the context of favorable microbiota-immune interactions [7].

Importantly, the study implemented machine learning (SVM and RF) approaches, integrating microbial and immune-related features to predict pCR with high accuracy. These models identified several microbiota species as key predictive features, including *P. pulmonicola*, *B. melitensis* and *Bacillus sonorensis* [7].

Despite these promising findings, Kim et al. recognized some limitations. The lack of functional of the identified microbial taxa precluded mechanistic insights into metabolite production and immune-modulatory pathways. In addition, the retrospective nature of the study limits causal inference and highlights the need for prospective validation in independent cohorts [7].

The study by Ransohoff et al. (2023) demonstrated that cumulative antimicrobial exposure, quantified by the total number of prescriptions and the number of unique antimicrobial agents per month, was significantly associated with reduced OS and BCS, independent of cancer stage, treatment modality and neutrophil count [30]. This association was sustained for up to 3 years following diagnosis, corresponding to the period of highest recurrence risk in early-stage TNBC.

Additionally, ALC emerged as an important mediating factor, as lymphopenia occurred more frequently among antimicrobial-exposed patients and was independently associated with worse clinical outcomes. Marginal structural modelling further suggested that antimicrobial-induced lymphopenia may compromise peripheral antitumor immunity, thereby affecting both immune surveillance and therapeutic efficacy [30]. These findings are consistent with previous evidence supporting the prognostic relevance oflymphocytes in TNBC. Higher peripheral lymphocyte counts have been associated with increased TIL density and improved response to neoadjuvant chemotherapy, whereas low ALC has been associated to early recurrence and increased mortality [55,56,57].

Mechanistically, antimicrobial agents are well-established disruptors of the gut microbiota, a key regulator of immune homeostasis. Antimicrobial exposure can result in reduced microbial diversity and depletion of immunostimulatory taxa, including as *Akkermansia muciniphila* and *Faecalibacterium prausnitzii*, both of which have been associated with enhanced responses to ICB in other tumor types. Furthermore, microbiota disruption may lead to reduced production of microbiota-derived metabolites and impaired antigen presentation and cytokine signaling, further compromising antitumor immune responses [46,47,48].

Although the study was only able to assess TIL in a limited subset of patients (*n* = 53) baseline stromal TIL density was confirmed as being significantly associated with pCR to chemotherapy. However, antimicrobial exposure was not significantly associated with either TIL density or pCR status within this limited cohort [30].

Despite the absence of ICB therapy in the analyzed population, these findings remain highly relevant to current clinical practice, particularly following the incorporation of pembrolizumab into neoadjuvant treatment regimens for early TNBC [58]. The efficacy of ICB relies on robust T cell mediated immune responses and a pre-existing inflamed tumor microenvironment, both of which are modulated by the gut microbiome. Studies in melanoma, non-small cell lung cancer (NSCLC) and renal cell carcinoma (RCC) demonstrated that antibiotic exposure before or during ICB therapy is associated with reduced treatment efficacy, an effect largely attributed to antibiotic-induced disruption of the gut microbiota [59,60,61,62].

The authors recognised that the small patient subset available for TIL and immune profiling limited the depth of mechanistic interpretation. Furthermore, the retrospective and observational design of the study precludes definitive conclusions regarding causality [30].

Consistent with these observations, a systematic review of clinical trials demonstrated that pembrolizumab significantly improved outcomes in patients with early-stage TNBC compared with placebo, irrespective of PD-L1 status. In advanced-stage breast cancer, pembrolizumab showed comparable efficacy to single-agent chemotherapy while offering a more favorable safety profile [6].

Chen et al. (2025) demonstrated that intratumoral microbiota, when integrated with MRI-derived radiomic features, can accurately predict response to NACI [31].

A key finding was that tumors achieving pCR exhibited a significantly higher intratumoral microbial burden, in line with preclinical evidence suggesting that specific microbial populations may function as local immune adjuvants, thereby enhancing antitumor immune responses [31,63,64,65].

Using scRNA-seq, the authors showed that microbial enrichment within tumors was associated with substantial remodelling of the TME. Bacteria-enriched tumors demonstrated increased proportions of FOLR2+ macrophages, a phenotype associated with antigen presentation and phagocytic activity, alongside a reduction in SPP1+ macrophages, which have been associated with immunosuppression and poor responsiveness to immunotherapy [31]. This immune remodeling suggested the hypothesis that intratumoral microbiota contribute to a more immune-permissive TME, potentially enhancing the efficacy of both cytotoxic and immune-mediated therapies. The inverse correlation between microbiota load and SPP1+ macrophage abundance further strengthens the biological plausibility of this association. Similar microbiota–immune patterns have been reported in other malignancies, including melanoma and non-small cell lung cancer, where beneficial microbial taxa correlate with increased immune cell infiltration [66,67,68,69].

Collectively, these findings suggest that local microbial communities may influence responsiveness to chemoimmunotherapy, potentially by enhancing antigenicity or modulating local immune activation [31].

Regarding the limitations of the study, it was conducted in a single-center cohort with a relatively small sample size, limiting generalizability. Additionally, microbiome characterization relied primarily on bulk 16S sequencing and surrogate markers, which may fail to capture relevant compositional or functional microbial differences. Finally, the contribution of the systemic microbiome in shaping the TME was not explored [31].

Yuan et al. (2021) provided insights into the potential convergence of hormonal modulation and immune checkpoint inhibition, possibly acting in synergy with the microbiome to enhance therapeutic efficacy in TNBC [32]. Although the primary objective of the trial was to assess the safety and efficacy of this dual-agent strategy, the exploratory microbiome analyses contribute meaningfully to the understanding of host-tumor-microbiota interactions in immunologically refractory TNBC subtypes [32].

In longitudinal analyses of the gut microbiome, while statistically significant associations were not observed, probably due to limited sample size, consistent trends emerged. Patients who experienced clinical benefit harbored gut microbial communities enriched in *Bacteroides* and *Alistipes*, taxa previously associated with improved responsiveness to ICB in melanoma, NSCLC and RCC [32,70,71,72]. These taxa are known to exert immunostimulatory effects through several mechanisms, including induction of dendritic cell maturation and antigen presentation; production of short-chain fatty acids, that promote Th1 polarization and IFN-γ secretion; and maintenance of gut epithelial barrier integrity, thereby limiting systemic inflammation and the release of immunosuppressive cytokine [73,74].

This study highlights the potential existence of microbiome-hormone-immune axis in cancer therapy. AR signaling is known to exert immunosuppressive effects, including dampening of type I interferon pathways and suppression of MHC expression [75]. Consequently, AR antagonism with enobosarm may enhance tumor antigen visibility and facilitate immune checkpoint blockade (ICB) efficacy when combined with pembrolizumab [32]. Simultaneously, a favorable gut microbiome composition may potentiate this effect by strengthening both innate and adaptive immune responses, suggesting that the therapeutic efficacy f ICB in AR-positive TNBC may depend on the interplay between endocrine signaling and microbiota composition. Although the microbiome analysis in this study was exploratory, its inclusion is highly relevant. It supports the concept that even TNBC molecular subtypes traditionally considered less immunogenic may be influenced by host-modifiable factors such as the gut microbiome. This influence may be particularly important in therapeutic contexts aimed at restoring or enhancing immune surveillance through systemic immunomodulatory strategies [32].

Nevertheless, several limitations must be acknowledged. These include the small sample size, absence of a comparator arm, and early termination of the trial due to drug availability, all of which limit the robustness of the conclusions. In addition, PD-L1 expression and TMB were not used as stratification variables. Moreover, the lack of tumor-associated microbiome profiling precludes direct assessment of interactions between gut microbial communities, and the tumor immune microenvironment remains inferential [32].

Complementary evidence from murine models provides important *in vivo* evidence for the impact of microbiome-targeted interventions on tumor behavior and immune modulation. The study by Yang et al. (2025) demonstrated that modulation of the gut microbiota through orally administered plant-derived extracellular vesicles significantly enhanced the antitumor efficacy of both ICB and NACT in a murine TNBC model [33].

This preclinical investigation identified convergent immunological and microbial mechanisms that reshaped TME. PGEV administration was associated with a favorable remodeling of the TME alongside beneficial alterations in intestinal microbial composition. Collectively, these findings align with an expanding body of evidence suggesting that dietary and natural product-derived agents can function as microbiome modulators, capable of sensitizing tumors to systemic anticancer therapies [33,76,77,78].

Additionally, these findings were mechanistically supported by extensive remodeling of the TME, characterized not only by increased infiltration of CD8^+^ T cell and increased IFN-γ expression but also a pronounced shift in macrophage polarization. PGEV treatment reduced the prevalence of immunosuppressive M2-like TAMs and promoted polarization toward an M1-like phenotype, as evidenced by decreased expression of CD206, a canonical M2 marker, and increased expression of iNOS, indicative of pro-inflammatory M1 macrophage activity. This macrophage repolarization is crucial in TNBC, where M2-TAM are well established as drivers of immune suppression and tumor progression [33,79,80].

In parallel, PGEV administration significantly reduced the abundance of Treg cells, reflected by decreased Foxp3+ cell frequencies within the tumor tissue [33]. Given the central role of Treg in facilitating immune evasion through suppression of cytotoxic CD8^+^ T cell function and inhibition of antigen-presenting cells [81], their reduction, in combination with MDSCs depletion and macrophage repolarization, suggests a broad reprogramming of the immunosuppressive TME toward a more immunostimulatory state. This immune reprogramming was associated with enhanced therapeutic efficacy of both ICB and NACT [33].

Importantly, these intratumoral immune effects were accompained by significant alterations in the gut microbiota, assessed through 16S rRNA sequencing. PGEV treatment increased α-diversity and induced compositional changes, including an increased relative abundance of *Lactobacillus* and the phylum *Firmicutes* and a reduction in *Bacteroides* and *Proteobacteria*. These findings are biologically relevant, as *Lactobacillus* species have been linked to improved epithelial barrier integrity and enhanced T cell priming, whereas *Proteobacteria* are frequently associated with pro-tumorigenic inflammation and immune dysfunction [33,82,83]. Although specific microbial metabolites were not quantified, the observed microbial restructuring may influence systemic immune responses through the production of immunomodulatory metabolites.

An important feature of this study is the combined treatment design, which evaluated PGEV in combination with anti-PD-1 immunotherapy and paclitaxel-based chemotherapy. PGEV co-administration enhanced the efficacy of both therapeutic modalities, supporting their potential role as an immunomodulatory adjuvant. PGEV appeared to reduce barriers to immune activation, namely MDSCs, Tregs and M2-TAMs, while ICB and NACT, respectively promote immune reinvigoration and immunogenic tumor cell death [33].

Despite the promising results, some limitations must be acknowledged including the absence of functional experiments to isolate the individual contributions of specific immune cell populations or microbial alterations, as well as the lack of metabolomic profiling to characterize the functional consequences of the observed e microbial shifts [33].

Pingili et al. (2021) demonstrated that, in the context of obesity and tumor burden, the systemic immune landscape is primed toward a potent immunosuppressive phenotype [34]. In obese tumor-bearing mice, a marked increase of splenic MDSCs (sMDSCs) was observed, encompassing both monocytic (M-sMDSCs) and polymorphonuclear (PMN-sMDSCs) subsets. These cells exhibited increase expression of MHC class II and PD-L1, indicative of enhanced immunosuppressive capacity. Additionally, obesity alone did not significantly affect sMDSCs levels in tumor-free mice; however, in the presence of a tumor, obesity appeared to amplify immunosuppressive signalling, creating a highly permissive environment for tumor immune evasion [34]. Similarly, obesity promoted a significant expansion of M2-like macrophages, an effect that was primarily evident in tumor-bearing mice.

Anti-PD-1 immunotherapy demonstrated a robust ability to reverse obesity-associated immune suppression. Treatment efficacy restored both systemic and intratumoral immune activity in obese mice, resulting in a significant reduction in tumor progression [34]. Within the TME, anti-PD-1 therapy induced a substantial increase in tumour-infiltrating DCs (tDCs), which are essential for antigen presentation and T cell priming. Although the overall frequency of TAM remained largely unchanged, treatment shifted macrophage polarization toward a pro-inflammatory phenotype, increasing M1-like TAMs while reducing M2-like TAMs, thereby favorably altering the M1/M2 ratio toward antitumor immunity [34].

The gut microbiome emerged as a key modulator of response to anti-PD-1 therapy in murine models of obesity-associated breast cancer. Diet-induced obesity, achieved through a high-fat diet, resulted in pronounced alterations in the relative abundance of specific bacterial taxa and overall microbial community structure [34]. These findings are consistent with a growing body of evidence indicating that microbial composition critically influences immunotherapy efficacy, particularly in metabolically dysregulated conditions such as obesity, where systemic inflammation and immune dysfunction are prevalent [84,85].

In the JE, obesity was associated with an increased relative abundance of genera such as *Enterococcus* and *Lactobacillus*, along with a reduction in *Bifidobacterium* and *Allobaculum*. Similarly, in the EC compartment, obesity resulted in enrichment of *Clostridia* and *Gammaproteobacteria*, with diet identified as the primary determinant shaping microbial community composition. Tumor presence alone was associated to a reduction in microbial abundance in both the ceca and jejunal communities and was further associated with a distinct separation in β-diversity within the EC between tumor-free and tumor-bearing mice [34]. Collectively, these microbial alterations are consistent with obesity-associated dysbiosis, which has been previously linked to systemic inflammation, increased intestinal permeability and impaired antitumor immune responses [86,87,88].

Administration of anti-PD-1 therapy induced notable changes in both microbial diversity and taxonomic composition. In the JE, treatment was associated with a reduction in α-diversity, whereas in the EC ecosystem a modest trend toward increased α-diversity was observed. Importantly, beneficial microbial taxa including *Akkermansia* and *Bifidobacterium* were consistently enriched following anti-PD-1 treatment in both lean and obese mice, consistent with clinical observations in human immunotherapy responders [34,89,90,91].

Conversely, bacterial taxa previously enriched in tumor-bearing animals, such as *Enterobacteriaceae*, *Clostridiaceae*, *Sutterella*, *Mucispirillum* and *Bacteroidales*, were significantly reduced following anti-PD-1 therapy in obese mice. In contrast, microbial genera that were suppressed in the presence of tumors, including *Lactobacillus*, *Bilophila*, *Dorea*, *Ruminococcus* and *Rikenellaceae* family were partially restored after treatment [34]. Notably, no shared taxa were downregulated by anti-PD-1 therapy across both lean and obese mice, suggesting that immunotherapy-induced modulation of the git microbiota is highly dependent on host metabolic status and follows a dichotomous regulatory pattern.

A positive statistically significant correlation between tumor volume and *Enterobacteriaceae* abundance in the EC of obese tumor-bearing mice was reported, and this bacterial family was notably reduced following anti-PD-1 treatment. In contrast, negative correlations with tumor size were observed for *Coriobacteriaceae*, *Bifidobacterium* and *Allobaculum* [34].

Functional interference of cecal microbial communities indicated that anti-PD-1 therapy was associated with upregulation of metabolic pathways involved in ion-coupled transport, DNA replication, oxidative phosphorylation, glycolysis and pyruvate, arginine and proline metabolism. In contrast, pathways involved in amino acid metabolism, methane metabolism, starch and sucrose metabolism were comparatively downregulated [34].

Despite the compelling results, several limitations of this study should be acknowledged. These include the absence of survival analysis, which limits conclusions regarding long-term therapeutic benefit, and the lack of functional microbiota experiments to establish causality. In addition, the contribution of microbiota-derived metabolites to the observed antitumor effects was not investigated [34].

The study by Kubota et al. (2023) investigated the gut microbiome as a therapeutic delivery platform for metabolic intervention in TNBC, using an engineered strain of *Escherichia coli* JM109, expressing recombinant methioninase [35]. This genetic modification enabled sustained enzymatic depletion of circulating methionine through stable colonization of the gastrointestinal tract. As a result, systemic methionine levels were significantly reduced, leading to a significant suppression of tumor growth in a TNBC orthotopic mouse model [35].

Methionine is a critical metabolic substrate required for methylation reactions, nucleotide synthesis and redox homeostasis in rapidly proliferating tumor cells. TNBC, in particular, exhibits a high degree of methionine dependence. Previous studies have demonstrated that methionine restriction, whether via diet or enzymatic depletion, impairs tumor cell proliferation and increases susceptibility to metabolic stress [92,93,94]. Consistent with these findings, the present study confirms that microbiome-mediated, *in situ* methionine depletion effectively recapitulated the antitumor effects of methionine restriction, reducing both tumor volume and weight without inducing systemic toxicity or affecting host body weight [35].

Mechanistically, methionine restriction reduces intracellular levels of S-adenosylmethionine (SAM), a central methyl donor in epigenetic regulation. Reduced SAM availability promotes global hypomethylation, disrupting gene expression programs essential for tumor cell survival, particularly in metabolically vulnerable subtypes such as TNBC. Additionally, methionine deprivation can compromise redox balance by limiting glutathione synthesis, increasing oxidative stress and promoting apoptotic signaling pathways, contributing to tumor growth arrest and cytotoxicity [95,96,97].

In addition, methionine metabolism has been implicated in the regulation of antitumor immunity through its effects on T cell activation, epigenetic remodeling and tumor antigen presentation. Methionine availability influences histone methylation in CD8^+^ T cells, modulating their differentiation into effector cells [98,99,100]. Emerging evidence further indicates that microbial regulation of amino acid metabolism can influence systemic cytokine profiles, dendritic cell activation and the expression of immune checkpoint ligands, such as PD-L1, establishing a mechanistic link between gut microbial metabolism and immune regulation [95,96,97].

Furthermore, the use of engineered commensal bacteria as vehicles for metabolic intervention highlights the therapeutic potential of the microbiome as a programmable platform capable of modulating host metabolic pathways. Engineered *E. coli* strains such as JM109 are generally well tolerated within the gut can be precisely designed to deliver therapeutic enzymes, metabolites, or signaling molecules. This strategy represents an emerging paradigm at the interface of synthetic biology and cancer therapy, enabling sustained and localized metabolic modulation through microbial colonization [35].

Lastly, some limitations that must be acknowledged include the absence of detailed immune profiling, which restricts insight into how microbiome-based interventions may interact with ICB. In addition, the limited scope of microbiota analysis precludes the identification of potential off-target or broader ecological effects on the gut microbiota [35].

The study by Manni et al. (2023), provides important evidence supporting the role of the gut microbiome in shaping the therapeutic response to chemotherapy in TNBC [36]. Specifically, the authors demonstrated that quercetin, a dietary polyphenol with known prebiotic properties, enhanced the antitumor efficacy of CTX in a murine TNBC model. Although quercetin alone did not exert a direct antitumor effect, its co-administration with CTX resulted in a significantly greater reduction in tumor volume compared with CTX monotherapy. These findings suggest that interactions between the host microbiota and the chemotherapeutic agent may partially underline the observed synergistic effect [36].

Quercetin supplementation, either alone or in combination with CTX, induced significant alterations in gut microbiome composition. Quercetin-treated mice displayed increased microbial diversity and enrichment of beneficial taxa, including *Lachnospiraceae*, *Ruminococcaceae* and *Akkermansia* which have previously been associated with immunostimulatory profiles and improved responses to immunotherapies and cytotoxic agents [36,101,102,103,104]. In contrast, CTX monotherapy was associated with reduced microbial diversity and an increased relative abundance of potentially less favorable bacterial families, such as *Bacteroides* and *Muribaculaceae*. These microbial changes were accompanied by shifts in predicted microbial metabolic functions, notably an enrichment in pathways related to SCFAs biosynthesis in the quercetin-treated groups [36]. Accordingly, elevated fecal levels of SCFAs, particularly butyrate and propionate, were observed in mice receiving quercetin in combination with CTX. SCFAs are known modulators of host immunity, influencing both both intestinal and systemic antitumor responses through mechanisms such as enhancement of dendritic cell function, promotion of memory T cell differentiation and suppression of regulatory T cell function [105,106,107,108].

Immunologically, CTX monotherapy increased intratumoral infiltration of CD8^+^ CTL, IFN-γ producing lymphocytes and granzyme B expression, consistent with its established immunomodulatory effects [36]. Importantly, the addition of quercetin significantly amplified these effects, leading to increased infiltration of CD4^+^ T cells and central memory T cells, as well as a reduction in immunosuppressive populations, including Treg cells and MDSCs. These findings suggested that microbiota modulation, via quercetin, may potentiate CTX-induced activation of adaptive immunity while concurrently mitigating immunosuppressive components of the TME [36]. These observations are of particular relevance in the context of ICB, as several taxa enriched in the quercetin plus CTX group, most prominently *Akkermansia* and *Lachnospiraceae*, have been consistently associated with improved clinical response to PD-1/PD-L1 inhibitors [101,102,103,104].

The complementary effects of quercetin on both microbial and immune compartments support a dual role in treatment potentiation, mediated through enhanced microbial metabolite production and immune cell priming.

Nevertheless, several limitations of the study must be acknowledged. Notably, the experimental design did not include causal microbiome validation approaches, such as microbiota depletion or fecal microbiota transplantation, limiting the ability to definitively attribute the observed effects to specific microbial changes. Moreover, the short treatment duration, together with the absence of comprehensive toxicity assessments and blinding procedures, constrains the generalizability and translational relevance of the findings [36].

The study by Bawaneh et al. (2022) provides further evidence that the presence and composition of the intestinal microbiota critically influence the antitumor efficacy of doxorubicin in TNBC [37]. Using a combination of antibiotic-induced microbita depletion, germ-free mouse models and FMT, the authors demonstrated that the therapeutic efficacy of doxorubicin is markedly attenuated in the absence of a functional microbiota and can be partially restored following microbial reconstitution. These results reinforced the growing recognition of microbiomes as a critical determinant of chemotherapeutic responsiveness in solid tumors, including breast cancer [37].

Mechanistically, the study provides several lines of evidence suggesting that the microbiota enhances doxorubicin efficacy through modulation of the antitumor immune landscape. Specific pathogen-free mice with intact microbiota exhibited robust tumor control in response to doxorubicin, in, accompanied by increased intratumoral infiltration of CD8^+^ cytotoxic T lymphocytes and elevated expression of granzyme B and IFN-γ [37]. These immune signatures were substantially diminished in antibiotic-treated and germ-free mice, suggesting that microbiota depletion compromises the ability of doxorubicin to elicit effective adaptive immune responses within the TME. Importantly, FMT was able to restore CD8^+^ T cell infiltration and partially rescue tumor suppression, further implicating microbial signals as facilitators of immune-dependent chemotherapy efficacy [37].

The observed immunological alterations were corroborated by RNA sequencing analyses, which revealed transcriptional downregulation of genes involved in interferon signaling and antigen processing in tumors from microbiota depleted mice. In contrast, tumors from SPF and FMT-reconstituted mice exhibited enriched expression of immune-related pathways, indicating preservation or reactivation of antitumor immune programs in the presence of an intact and functional microbiome [37]. These findings are consistent with previous reported data in other tumor models, in which microbiota-derived signals, including microbial metabolites, toll-like receptor ligands and microbiota-associated molecular patterns (MAMPs), have been shown to enhance antigen presentation, modulate myeloid cell activation and promote CD8^+^ CTL function during cytotoxic chemotherapy [100,101,102,103,104,105,106,107,108,109,110,111].

These results carry direct relevance for ICB strategies, as CD8^+^ T cells activity, IFN-γ signaling and efficient antigen presentation are central determinants of ICB efficacy Moreover, prior studies have demonstrated that microbiota depletion similarly impairs responses to PD-1 blockade in murine models, suggesting the existence of a shared immunological pathways through which the microbiome modulates responsiveness to both chemotherapy and immunotherapy [112,113].

Notably, these findings also highlight the microbiome’s potential role in overcoming resistance in TNBC, a tumor subtype characterized by an immunologically cold microenvironment and heterogeneous responses to chemotherapy. However, several limitations should be considered. The authors noted the potential confounding effect of menopause status on the gut microbiome composition. Additionally, host-related factors were not uniformly controlled, limiting reproducibility and the broader translational applicability of the results [37].

The following study integrates human-derived datasets with murine models, providing a translational framework for interpreting the biological relevance and *in vivo* efficacy of microbiome-mediated interventions.

The study by Wang et al. (2022) offers highly significant and mechanistically grounded insights into the role of the microbiome in shaping antitumor immunity and response to ICB in TNBC [39]. Unlike observational studies, this study combined multi-omics analyses of human tumor samples with functional validation in syngeneic murine models, thereby establishing a translational link between microbial metabolic activity and immunotherapeutic outcomes [38].

One of the most important findings of this study was the identification of the microbiota-derived metabolite TMAO as a central mediator of ICB efficacy in TNBC. TMAO levels were significantly enriched in human tumors exhibiting an immune-activated phenotype and positively correlated with increased of CD8^+^ cytotoxic T lymphocytes, elevated IFN-γ expression and improved progression-free survival in patients receiving anti–PD-1 therapy [38]. These associations were mechanistically validated *in vivo* using syngeneic murine TNBC models, in which systemic or intratumoral administration of TMAO significantly enhanced the antitumor efficacy of anti-PD-1 therapy.

Mechanistically, TMAO promoted GSDME-mediated pyroptosis, a highly immunogenic form of programmed cell death. This process triggered a local inflammatory cascade characterized by the release of damage-associated molecular patterns (DAMPs), including IL-1β and IL-18, which are known to promote the recruitment and activation of immune effector cells within the TME [38,114,115]. Importantly, TMAO-induced pyroptosis resulted in increased infiltration and functional activation of CD8 +CTL cells, as evidenced by elevated IFN-γ production and pronounced tumor regression. Notably, combined treatment with TMAO and anti–PD-1 therapy achieved significantly greater tumor control than anti–PD-1 monotherapy [38]. This synergistic effect was abolished following CD8^+^ T cells depletion or pharmacological inhibition of PERK signaling, underscoring the central role of TMAO-induced pyroptosis in mediating therapeutic benefit [38]. These findings suggest that TMAO may function not only as a biomarker of immunotherapy responsiveness, but also as a potential therapeutic adjuvant capable of sensitizing TNBC tumors to ICB [38].

Additionally, the study established a link between dietary modulation of the microbiome and immunotherapeutic efficacy. Mice fed a choline-enriched diet, a TMAO precursor, demonstrated significantly increased intratumoral TMAO levels and enhanced responses to PD-1 blockade. Conversely, co-administration of DMB, an inhibitor of microbial TMAO synthesis, abolished these effects, confirming the requirement for an intact microbial enzymatic pathway. By promoting immunogenic cell death and amplifying CD8^+^ T cells responses, TMAO represents a mechanistic link between microbial metabolism and host antitumor immunity [38]. These findings highlight the potential of dietary interventions and microbiota-targeted strategies to enhance immunotherapy efficacy in TNBC.

However, some limitations warrant consideration. Clinical validation of TMAO as a predictive biomarker was based on a small subset of patients receiving anti-PD-1 therapy (*n* = 12), limiting statistical power. Furthermore, microbial taxonomy was not directly manipulated, and causal associations between specific bacterial taxa and TMAO production were inferred rather than experimentally confirmed [38].

The integrated use of *in vitro* cell lines and murine models in the following study provides complementary insights into both the molecular and systemic effects of the interventions.

In the study by Zhang et al. (2024), mechanistically relevant evidence was presented, supporting a broader conceptual framework in which microbial products modulate the tumor immune microenvironment and metastatic behavior in TNBC [39]. Specifically, the study demonstrated that ΔA146Ply, a detoxified form of pneumolysin, a pore-forming toxin derived from *Streptococcus pneumoniae*, can inhibit metastasis in a TNBC murine model by modulating autophagy and macrophage polarization via mannose receptor (CD206-) mediated signaling.

This work strengthens the emerging link between microbiome, imme modulation and cancer therapy by employing a microbial-derived protein capable of engaging innate immune pathways and reshaping immune cell function within the tumor microenvironment [39]. Administration of ΔA146Ply significantly reduced lung metastases without affecting primary tumor growth, suggesting a selective inhibitory effect on metastatic dissemination rather than on tumor initiation or local progression. Mechanistically, the compound disrupted autophagic flux by inducing lysosomal dysfunction, as evidenced by the accumulation of autophagosomes and impaired autophagosome-lysosome fusion. Importantly, these effects were dependent on mannose receptor-mediated uptake, underscoring the involvement of pattern recognition receptors commonly engaged by microbial ligands in the host immune system [39].

Additionally, the study reported notable immunomodulatory effects, particularly a phenotypic shift in TAMs from a M2-like (CD206+) to an M1-like (CD86+) state. This macrophage reprogramming following ΔA146Ply treatment is consistent with prior evidence demonstrating that the microbiome and its metabolites can influence macrophage phenotypes and, consequently, treatment outcomes [39,79,80]. The increased levels of proinflammatory cytokines such as tumor necrosis factor (TNF) α and IL-6 further reinforce the notion that ΔA146Ply promotes an immune-permissive microenvironment.

The engagement of innate immune signaling pathways, together with evidence of autophagy inhibition and TAMs repolarization, suggests mechanistic overlap with known microbiota-dependent immunological axes. Prior investigations have shown that microbial components such as lipopolysaccharides, flagellins and bacterial toxins can function as immune adjuvants, enhance antigen presentation and sensitize tumors to immune and cytotoxic therapies [116,117,118,119]. Therefore, ΔA146Ply may act as a microbial mimic, triggering pattern recognition receptors and modulating autophagy-immune interactions.

These findings support a broader hypothesis that microbials or microbial-derived agents could serve as adjuncts to conventional TNBC therapies by reprogramming the tumor microenvironment, particularly through macrophage-dependent mechanisms. Nonetheless, several limitations must be acknowledged. The study did not include direct analysis of the endogenous microbiome, nor did it evaluate ΔA146Ply in combination with standard-of-care therapies. Additionally, many of the reported associations remain correlative, limiting causal inference [39].

Complementing these *in vivo* findings, *ex vivo* organoid co-culture systems offer a valuable platform for mechanistic evaluation of microbiota-driven immunomodulation within a controlled tumor–immune environment.

The study by Shelkey et al. (2022) provides important preclinical insights into the role of microbiome-derived signals in modulating antitumor immunity and IBC efficacy [40]. Using a well-controlled *ex vivo* organoid co-culture model, the authors demonstrated that soluble factors derived from the fecal microbiota of untreated mice significantly enhanced CD8^+^ T cell activation, cytotoxicity and tumor cell apoptosis when combined with anti-PD-1 therapy. In contrast, microbiota-derived filtrates from antibiotic-treated mice failed to reproduce these immunostimulatory effects, underscoring the requirement for an intact microbiome to achieve optimal ICB responsiveness [40].

This study reinforces the emerging paradigm in which MAMPs, and microbial metabolites act as systemic immune modulators capable of shaping the tumor-immune interface. The experimental design enabled the isolation of microbiome-derived soluble factors by excluding live bacteria, thereby focusing on metabolite and MAMP-driven mechanisms [40]. Notably, the enhancement of anti-PD-1 mediated CD8^+^ T cell activity was accompanied by increased expression of effector molecules, including granzyme B and IFN-γ, and by elevated tumor cell apoptosis, as evidenced by caspase-3 activation. These findings are consistent with previous murine studies demonstrating that specific microbial taxa, such as *Bifidobacterium* and *Akkermansia*, or their metabolic products, supported T cell infiltration and improved response to ICB [89,90,91].

Mechanistically, these findings implicate microbiota-derived factors in the amplification of antigen-specific T cell responses within the TME. The observed increase in CD8^+^ T cell frequency and function in the presence of microbiota-intact filtrates is consistent with enhanced antigen presentation and T cell priming [40]. Importantly, this organoid-based model represents a valuable platform for dissecting microbiome-immune interactions in a controlled, physiologically relevant context. By enabling the evaluation of microbial signals in the absence of *in vivo* colonization, this approach supports the hypothesis that microbiome composition and function output are critical determinants of immunotherapy efficacy, including PD-1 blockade.

In the context of TNBC, a tumor subtype frequently characterised by low immunogenicity and limited response to ICB monotherapy, these results underscore the therapeutic potential of microbiome-informed interventions. By identifying microbiota-derived signals capable of enhancing T cell-mediated tumor cell killing, this study reinforces the rationale for integrating microbial modulation strategies, such as FMT, dietary modulation or microbial metabolite delivery, into combination regimens for TNBC.

Despite the accumulating preclinical evidence, microbiome-ICB interactions have not yet been rigorously studied in TNBC-specific clinical trials. Moreover, limitations within the existing literature, including heterogeneity in microbiome sampling methodologies and confounding variables such as diet, antibiotic exposure and unreported concomitant therapies, remain the main limitations of the interpretation and translation of findings in this field [7,28,29,30,120,121,122].

## 5 Conclusion, Limitation and Future Perspective

This systematic review underscores the microbiome as an emerging and clinically relevant determinant of therapeutic response in TNBC. As treatment strategies continue to evolve with the incorporation of ICB into NACT regimens, there is an increasing need to identify modifiable biological factors that can optimize patient outcomes. In this context, the composition, diversity and functional output of the microbiome, both intestinal and intratumoral, represent promising targets for therapeutic stratification and intervention.

Accumulating evidence suggests that the gut microbiome influences TNBC outcomes through the priming and coordination of innate and adaptive immune responses. Higher microbial diversity has been associated with a more immunologically permissive landscape, characterized by enhanced dendritic cell maturation, T cell activation and sustained cytokine signaling, all of which are critical for maximizing the efficacy of cytotoxic chemotherapy and ICB. Additionally, specific commensal taxa and metabolites, including short-chain fatty acids, inosine and other immunoregulatory compounds, appear to support cytotoxic T-lymphocyte expansion, maintain immune homeostasis and preserve mucosal integrity during treatment.

In parallel, the tumor-resident microbiome has been implicated in shaping the TME. Specific microbial profiles within breast tumors have been correlated with increased infiltration of effector immune cells such as CD8^+^ T cells, NK cells and M1 macrophages, whereas other microbial signatures are associated with immunosuppressive phenotypes characterized by regulatory T cells and M2 macrophages. These microbial-immune interactions within the TME are particularly relevant in TNBC, a subtype characterized by relatively high immunogenicity and responsiveness to immune-modulating therapies.

Beyond immunotherapy, the microbiome also influences sensitivity to chemotherapy, through modulation of immunogenic cell death, tumor antigen release and downstream immune activation. By facilitating the recognition and processing of dying tumor cells and promoting efficient cross-priming of T cells, microbiota may enhance the potential of chemotherapy to induce durable antitumor immune memory. In the context of combined chemoimmunotherapy, the microbiome may therefore function as a synergistic amplifier, ensuring that cytotoxic stress is effectively translated into a robust and sustained anti-tumor response.

Conversely, microbiome disruption, particularly through antibiotic exposure, has been associated with poorer clinical outcomes, including reduced overall and disease-specific survival. This effect is thought to result from the depletion of beneficial microbial taxa that supports immune competence and regulate systemic inflammation. Importantly, the influence of the microbiome extends beyond the gut, as emerging evidence suggests that local breast tissue microbiota also modulates gene expression, immune cell recruitment and epigenetic regulation, thereby further shaping tumor behavior and therapeutic responsiveness.

Collectively, these findings support the integration of microbiome-based therapies into TNBC treatment paradigms. Microbiome profiling holds promise as a non-invasive biomarker for patient stratification, while targeted interventions, such as prebiotics, probiotics, dietary modulation or fecal microbiota transplantation, may represent adjunctive strategies to enhance therapeutic efficacy. Moreover, the microbiome’s ability to modulate both systemic and local immune responses suggests a potential role in mitigating treatment resistance and reducing disease recurrence, thereby improving the durability of therapeutic benefit in TNBC.

Nevertheless, the current body of evidence is largely derived from preclinical or small, retrospective clinical cohorts, which impose several limitations. These include substantial heterogeneity in microbiome sampling and sequencing methodologies, inadequate control of confounding factors (such as, diet, antibiotics, comorbidities) and a lack of prospective validation in randomized clinical trials. In addition, the dynamic evolution of the microbiome throughout the course of treatment remains insufficiently characterized and warrants further investigation.

To fully elucidate the therapeutic potential of the microbiome in TNBC, future studies should prioritize longitudinal, multi-omics investigations that integrate microbiome profiling with immune and transcriptomic analyses. Functional studies are also needed to clarify causal associations between microbial metabolites and immune effectors; while well-designed clinical trials should evaluate microbiota-targeted interventions in combination with standard TNBC therapies, particularly chemo-ICB regimens.

In conclusion, emerging evidence indicates that the microbiome plays a relevant role in modulating pathological response in TNBC. Interactions between the microbiome, host immune response, and tumor microenvironment appear to influence sensitivity to systemic therapies, with potential implications for pathological response rates. A deeper understanding of these microbiome-host-tumor interactions may contribute to more personalized therapeutic strategies, guiding treatment selection and ultimately improving patient outcomes.

## Data Availability

Not applicable.

## Supplementary Materials

The supplementary material is available online at https://www.techscience.com/doi/10.32604/or.2026.074215/s1.

## Abbreviations

ALC	Absolute Lymphocyte Count
ANC	Absolute Neutrophil Count
AR	Androgen Receptor
AUC	Area Under the Curve
BCS	Breast Cancer-Specific Survival
BMI	Body Mass Index
CBR	Clinical Benefit Rate
CD	Cluster of Differentiation
CI	Confidence Interval
CTLs	Cytotoxic T Lymphocytes
CTX	Cyclophosphamide
DAMPs	Damage-Associated Molecular Patterns
DMB	3,3-Dimethyl-1-Butanol
CE	Cecum
ER	Estrogen Receptor
FF	Fecal Filtrates
FISH	Fluorescence *In Situ* Hybridization
FMT	Fecal Microbiota Transplantation
GEO	Gene Expression Omnibus
GF	Germ-Free
GSEA	Gene Set Enrichment Analysis
GM	Gut Microbiota
HER2	Human Epidermal Growth Factor Receptor 2
HR	Hazard Ratio
ICB	Immune Checkpoint Blockade
IHC	Immunohistochemistry
IL	Interleukin
IPTG	Isopropyl-D-Thiogalactopyranoside
IV	Intravenous
IFN-γ	Interferon-gama
JE	Jejunum
LPS	Lipopolysaccharide
MAMPs	Microbiota-Associated Molecular Patterns
MDSCs	Myeloid-Derived Supressor Cells
ML	Machine Learning
MRI	Magnetic Resonance Imaging
mTNBC	Metastatic Triple Negative Breast Cancer
NACI	Neoadjuvant Chemoimmunotherapy
NACT	Neoadjuvant Chemotherapy
NK	Natural Killer
NSCLC	Non-Small Cell Lung Cancer
ORR	Objective Response Rate
OS	Overall Survival
PBS	Phosphate-Buffered Saline
pCR	Complete Pathological Response
PCR	Polymerase Chain Reaction
PD	Phylogenetic Diversity
PD-1	Programmed Death 1
PD-L1	Programmed Death-Ligand 1
PFS	Progression-Free Survival
PGEVs	*Platycodon grandiflorum*-Derived Extracellular Vesicles
PLD	Pegylated Liposomal Doxorubicin
PR	Progesterone Receptor
PRISMA	Preferred Reporting Items for Systematic Reviews and Meta-Analyses
RCC	Renal Cell Carcinoma
RF	Random Forest
rMETase	Recombinant Methioninase
ROS	Reactive Oxygen Species
SAM	S-Adenosylmethionine
SARM	Selective Androgen Receptor Modulator
SCFAs	Short-Chain Fatty Acids
scRNA-seq	Single-Cell RNA sequencing
SPF	Specific Pathogen-Free
SVM	Support Vector Machine
TAMs	Tumor Associated Macrophages
TC	Tetracycline
TCGA	The Cancer Genome Atlas
TGF	Transforming Growth Factor
TILs	Tumor-Infiltrating Lymphocytes
TMAO	Trimethylamine N-oxide
TME	Tumor Microenvironment
TNBC	Triple Negative Breast Cancer
TNF	Tumor Necrosis Factor
